# Enhancing Evidence-Based Pharmacy by Comparing the Quality of Web-Based Information Sources to the EVInews Database: Randomized Controlled Trial With German Community Pharmacists

**DOI:** 10.2196/45582

**Published:** 2023-06-21

**Authors:** Jennifer Maria Alexa, Matthias Richter, Thilo Bertsche

**Affiliations:** 1 Department of Clinical Pharmacy Institute of Pharmacy, Faculty of Medicine, Leipzig University Leipzig Germany; 2 Drug Safety Center Leipzig University Hospital Leipzig University Leipzig Germany

**Keywords:** databases, electronic information, evidence-based pharmacy practice, evidence-based pharmacy, evidence-based practice, external evidence, health information quality, information tools, newsletter, online survey, pharmacist, self-medication counseling, self-medication, utilization

## Abstract

**Background:**

Self-medication counseling in community pharmacies plays a crucial role in health care. Counseling advice should therefore be evidence-based. Web-based information and databases are commonly used as electronic information sources. EVInews is a self-medication–related information tool consisting of a database and monthly published newsletters for pharmacists. Little is known about the quality of pharmacists’ electronic information sources for evidence-based self-medication counseling.

**Objective:**

Our aim was to investigate the quality of community pharmacists’ web-based search results for self-medication–related content in comparison with the EVInews database, based on an adjusted quality score for pharmacists.

**Methods:**

After receiving ethics approval, we performed a quantitative web-based survey with a search task as a prospective randomized, controlled, and unblinded trial. For the search task, participants were instructed to search for evidence-based information to verify 6 health-related statements from 2 typical self-medication indications. Pharmacists across Germany were invited via email to participate. After providing written informed consent, they were automatically, randomly assigned to use either web-based information sources of their choice without the EVInews database (web group) or exclusively the EVInews database (EVInews group). The quality of the information sources that were used for the search task was then assessed by 2 evaluators using a quality score ranging from 100% (180 points, all predefined criteria fulfilled) to 0% (0 points, none of the predefined criteria fulfilled). In case of assessment discrepancies, an expert panel consisting of 4 pharmacists was consulted.

**Results:**

In total, 141 pharmacists were enrolled. In the Web group (n=71 pharmacists), the median quality score was 32.8% (59.0 out of 180.0 points; IQR 23.0-80.5). In the EVInews group (n=70 pharmacists), the median quality score was significantly higher (85.3%; 153.5 out of 180.0 points; *P*<.001) and the IQR was smaller (IQR 125.1-157.0). Fewer pharmacists completed the entire search task in the Web group (n=22) than in the EVInews group (n=46). The median time to complete the search task was not significantly different between the Web group (25.4 minutes) and the EVInews group (19.7 minutes; *P*=.12). The most frequently used web-based sources (74/254, 29.1%) comprised tertiary literature.

**Conclusions:**

The median quality score of the web group was poor, and there was a significant difference in quality scores in favor of the EVInews group. Pharmacists’ web-based and self-medication–related information sources often did not meet standard quality requirements and showed considerable variation in quality.

**Trial Registration:**

German Clinical Trials Register DRKS00026104; https://drks.de/search/en/trial/DRKS00026104

## Introduction

### Background

The treatment of clinical complaints with over-the-counter (OTC) products plays a substantial role in health care [1]. Community pharmacists are often the first and only point of contact within the health care system for self-medication–related inquiries [2]. Self-medication counseling by pharmacists can contribute to a cost-effective, individualized, safe medical care on the patient level and consequently to a relief of the burden of the entire health care system [3,4]. External evidence is required for well-grounded consultations and a key factor within the 3-fold evidence-based pharmacy (EbPharm) concept, which derived from evidence-based medicine [5]. Apart from external evidence, the pharmacist’s practical experience and the patient’s preference should be considered when counseling [6,7]. However, a lack of information sources, time, and search skills has been identified as common barriers to EbPharm [8-18]. Consequently, various evidence-based information resources were introduced to reduce the evidence-to-practice gap. Among all information sources, electronic sources are of increasing importance for pharmacists [19,20]. The majority of information sources are however not targeted for community pharmacists, especially not in the context of self-medication counseling [21-29]. The EVInews project is an example for an electronic, evidence-based self-medication–related information platform for community pharmacists in Germany [30].

Little is known about the quality and the kind of electronic information sources that pharmacists use for evidence-based self-medication counseling. Information quality in the context of health is defined as a multidimensional concept that describes the fulfillment of individually predefined criteria [31,32]. Several attempts have been made to measure the quality of electronic information sources through validated instruments [33-39]. Standard categories of these instruments address an information source’s transparency, accuracy, completeness, and readability for instance [40]. These instruments help identify valid information among the increasing body of available electronic data. Previous instruments, as a limitation, were only used for patient-oriented websites.

To date, literature about the quality of pharmacists’ electronic information sources for self-medication counseling has been scarce. In particular, studies that investigate the quality based on appropriate standard quality criteria of such electronic information sources are missing.

### Study Objectives

Our aim was to investigate the quality of community pharmacists’ web-based search results for self-medication–related content when using the internet in comparison with the EVInews database, based on an adjusted quality score for pharmacists. Furthermore, this study attempted to explore what types of web-based sources are used for evidence-based self-medication counseling and how much time is needed under everyday practice conditions.

## Methods

### Study Design

A quantitative, unblinded, prospective randomized controlled trial including a web-based survey with an unblinded search task and a parallel study design was carried out between February 1, 2021, and April 30, 2021. The survey and data collection were conducted using SoSci Survey, Leiner DJ (version 3.1.06). Participating pharmacists were automatically assigned through the randomization function of the software in a 1:1 allocation ratio to either study group after giving their informed consent.

### Study Population and Setting

Community pharmacists across Germany within 5 different federal pharmacy chambers (Baden-Württemberg, Bavaria, Mecklenburg-Western Pomerania, Lower Saxony, and Saxony) were invited via an email to participate remotely and anonymously without any incentives. For this purpose, invitations containing the survey were sent via email to all community pharmacies that agreed to receive survey invitations and were listed in the email list of each federal pharmacy chamber. The completeness rate of the survey and time needed for completion were assessed through the SoSci Survey software. A reminder email was sent after 1 month if less than 15 participants within a pharmacy chamber were enrolled. Participants had to be licensed pharmacists to be included in the study.

### Study Groups

Participants were instructed to solely use either web-based information sources of their choice except for the EVInews database (web group) or exclusively the EVInews database (EVInews group). The EVInews group accessed the database via the link provided [41] and a test account.

### Survey Structure

The survey was divided into 3 main categories as follows: sociodemographic data, verification of 6 health-related statements with assigned information source type from 2 typical self-medication indications (search task), and self-evaluation of search skills and feedback.

### Search Task

Participants of both study groups were asked to verify 6 health-related statements from 2 typical self-medication indications (Table S1 in Multimedia Appendix 1) with a quote and the corresponding information source (search task). Of the 6 statements, 3 belonged to the self-medication indications recurrent herpes labialis and female androgenetic alopecia, respectively*.* The indications were chosen deliberately because they require specific knowledge but are nonetheless relevant for everyday practice, and content was available in the EVInews database.

A video and a short written summary were presented to all participants to inform them about the study protocol. The 3 statements of each indication were then displayed in an alternating order based on the software’s Rotate Blocks function to the participants. This was used to prevent learning effects and therefore a distortion of the results. Participants were then instructed to copy and paste their quote and link of the information source that proved or disproved the correctness of each statement in designated textboxes.

### The EVInews Database

The EVInews project was launched in 2017 and is based on the resolution of the Federal Union of German Associations of Pharmacists (ABDA) to help implement EbPharm into everyday practice. It consists of a website with general EbPharm information, monthly published newsletters, and a corresponding document-oriented database, which are free of charge. Once registered, users can access the database through a login. Independent pharmacists from the Department of Clinical Pharmacy at Leipzig University compose the newsletters with editorial assistance from AVOXA—Media Group German Pharmacist GmbH.

### Quality Score

An adjusted quality score was used to assess the information sources that were used for the search task. Therefore, a rating scheme for the evaluation of the search task was created (Table S2 in Multimedia Appendix 2).

### Rating Scheme

Rating scheme criteria were chosen and adapted based on validated quality scores and questionnaires reported in the literature [35-37] as well as previous findings [32,39,42-44]. The rating scheme consists of 18 nominal- to ordinal-scaled criteria that address predefined quality standards. We selected existing and added new criteria that were relevant for the search task. The rating scheme criteria were grouped together in the categories “transparency,” “quality of evidence,” and “usability” for the information sources and “accuracy” for the evaluation of the quotes. Each quote’s accuracy was evaluated subjectively with regard to logic, rationale, and comprehension and objectively based on a summative content analysis. Of all items, 12 had a dichotomous response format and 6 had an ordinal response format.

### Quality Assessment Procedure

Participants’ quotes and sources were independently evaluated by 2 evaluators (MR and JMA) based on the rating scheme and merged after analysis completion. A total median was calculated based on the 2 different evaluations. The maximum quality score for a quote and information source for all 6 statements accounted to 180 points (ie, all criteria were fulfilled). Each rater was requested to provide reasons for point allocation.

### Study Subgroups

**Figure 1:**
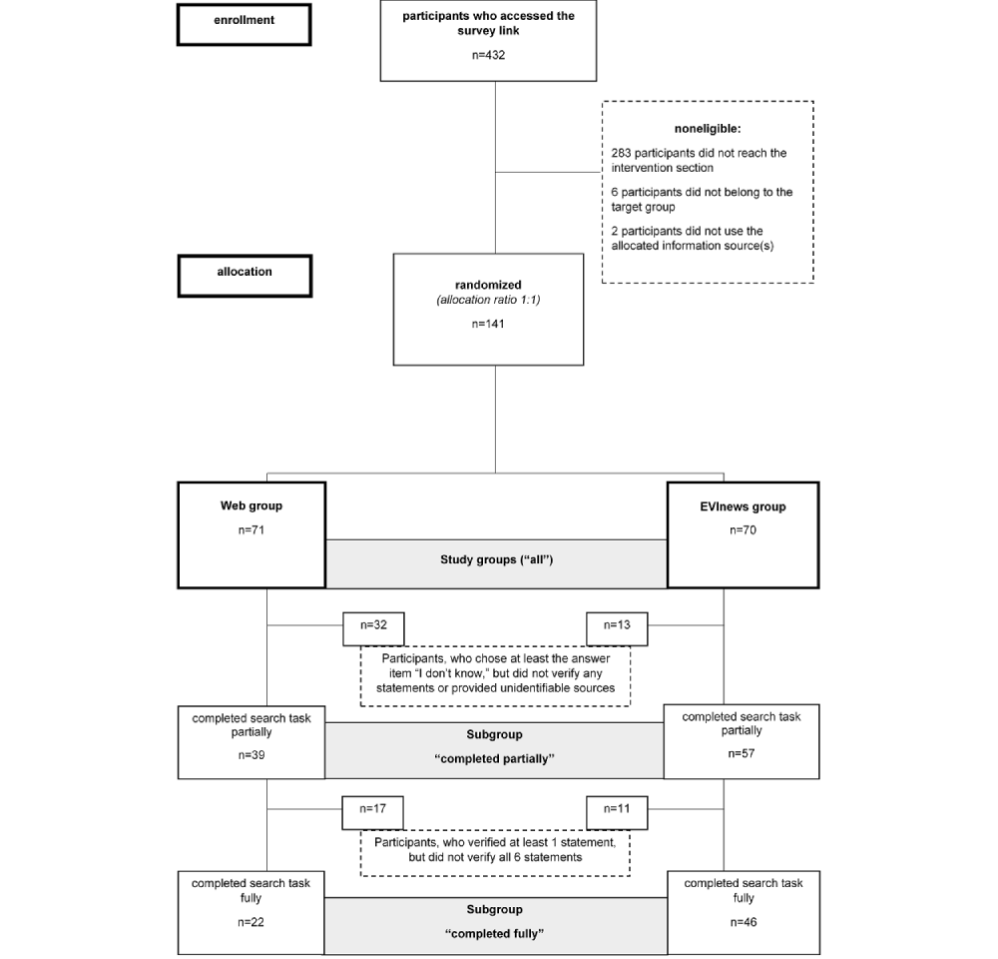
Participant flowchart modified according to CONSORT-EHEALTH (Consolidated Standards of Reporting Trials of Electronic and Mobile HEalth Applications and onLine TeleHealth) [45].

Because not all participants completed the search task of the survey, a subgroup analysis of these participants was performed as demonstrated in Figure 1. Participants who chose at least 1 answer option including “I do not know” within the search task were defined as “met the eligibility criteria (‘all’).” Participants who chose 1 answer option and verified at least 1 statement with a quote and source were defined as “completed search task partially (subgroup ‘completed partially’).” Participants who verified all 6 health-related statements were defined as “completed search task fully (subgroup ‘completed fully’).”

### General Study Procedure

**Figure 2:**
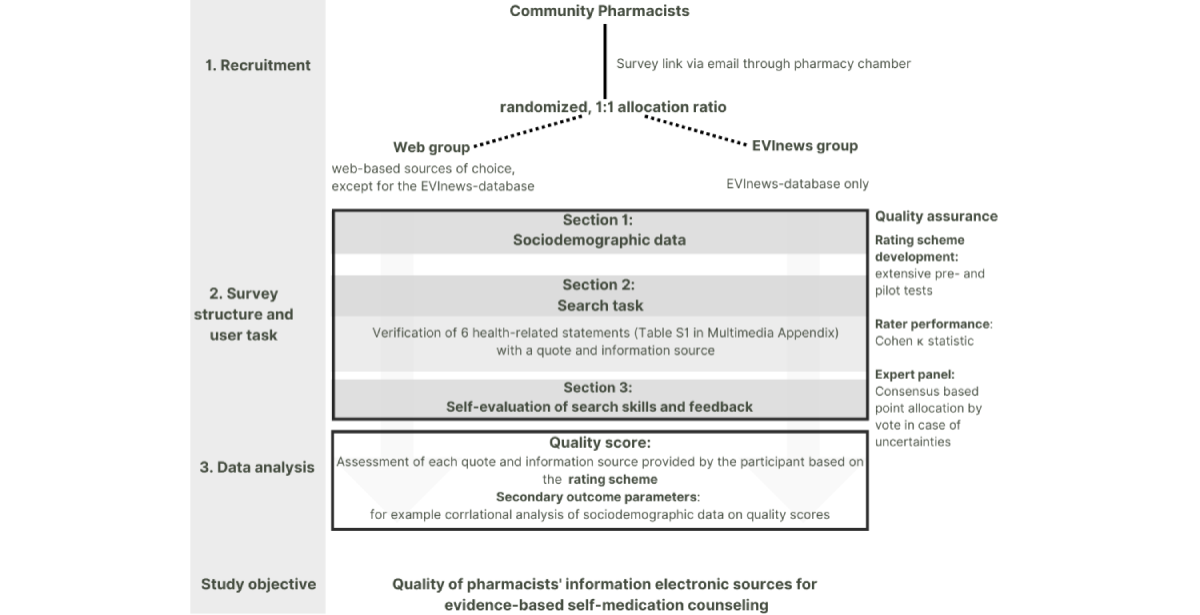
Study Protocol.

The study procedure is shown in Figure 2.

The survey consisted of 32 questions, which included close-ended and open-ended questions. Dichotomous, 4-point Likert scales and multiple-choice answer options were given. The level of measurement ranged from nominal- to ordinal-scaled data.

### Outcome Parameters

The primary outcome parameter was the difference in quality scores between the study groups. Secondary outcome parameters included a subgroup analysis, quality scores of the rating scheme categories of transparency, quality of evidence, and usability, the characterization of web-based information sources, a correlational analysis of sociodemographic characteristics and quality scores, a self-evaluation of search skills by the participants, and assessment of the time needed to perform the search task.

### Sample Size

The estimated sample size was calculated using the G*Power, software, Faul F, Erdfelder E, Buchner A, Lang AG (version 3.1). No information regarding the primary target distribution was available. Based on the results of a pilot test, a double-sided Mann-Whitney *U* test for nonparametric, independent samples was used a priori to determine the required sample size for a significant difference in quality scores. At a significance level of α=.05 and a CI of 95%, a sample size of at least 44 pharmacists per group would provide a power of 1 – β=.80. We estimated attrition to be 50% and consequently strived for a greater sample size.

### Data Analysis

Only data of participants who processed the search task and did not violate the study protocol were included in the data analysis. The analysis was performed using Excel (versions 2105 and 2016; Microsoft Corp) and SPSS Statistics (version 25 and 26; IBM Corp). Results for the primary target parameter (quality score) were expressed as median and first and third quartile (Q_25_/Q_75_). They were compared using a 2-sided Mann-Whitney *U* test for independent samples. The comparison of quality subscores was performed likewise with a Bonferroni correction. Descriptive statistics were used to summarize the responses. The Spearman rank correlation coefficients were calculated to investigate the relationship between quality scores and sociodemographic data.

### Quality Assurance Means

#### Survey and Quality Score Development

To ensure feasibility and exclude comprehension difficulties, pretests and pilot tests were conducted between October 26, 2020, and January 19, 2021. The pretests were carried out with 23 pharmacists. The first 12 pretests were executed using the think-aloud method, the remaining using the standard observation before the test. After a revision and adjustment of the survey and rating scheme, a pilot test with 37 pharmacists and advanced pharmacy students as pretesters was performed, resulting in minor modifications. Furthermore, to reduce subjective judgment on each quote’s suitability (item 4d within the rating scheme), 1359 relevant keywords in English and German were identified during the pretest phase and used for a summative content analysis. The frequency of these predefined keywords was measured through the Excel (Microsoft Corp) count function for each quote. To assess whether difficulties occurred during the completion of the search task, participants were asked to rate the clarity of this survey and give qualitative feedback if desired. Data generated by the pretest and pilot test were not included in this study.

#### Expert Panel

Quotes and sources that were not analyzable based on the rating scheme by at least one of the evaluators were presented to an expert panel in May 2021. The expert panel consisted of 4 pharmacists specialized or mostly in continuing education to become a pharmacist with a focus on drug information, who were not involved in the EVInews project. Consensus-based point allocation was ultimately done by vote for the remaining uncertainties.

#### Rater Performance

To examine whether differences in rater performance were present, interrater reliability was assessed using the Cohen κ statistic. The Cohen κ coefficient for each item of the rating scheme and subsequently for all rating scheme items was calculated and compared with the qualitative interrater reliability descriptors by Landis and Koch [46].

### Ethics Approval

Ethical approval for this study was granted by the ethics committee of the Medical Faculty of the Leipzig University (541/20-ek) on December 5, 2020.

## Results

### Participant’s Characteristics

The participant flowchart (Figure 1) depicts the participants who have been enrolled.

The participant’s characteristics are summarized in Table 1. Of all 141 participants, 110 (78.0%) were female and 31 (22.0%) were male. The median age interval was between 41 and 50 years (31/141, 22.0%) and more than half of all participants had more than 11 years of work experience (79/141, 56.0%). A reminder email was sent to the members of the Pharmacy Chamber of Saxony because of a low response rate in the first round.

A Fisher exact test revealed no significant sociodemographic differences between the 2 study groups concerning gender, age, and academic degree.

**Table 1:** Sociodemographic data of the study sample (N=141).

Characteristics		Web group (n=71)	EVInews group (n=70)
**Gender, n (%)**			
	Female	56 (79)	54 (77)
	Male	15 (21)	16 (23)
**Age (years), n (%)**			
	≤30	10 (14)	18 (26)
	31-40	22 (31)	20 (29)
	41-50	16 (23)	15 (21)
	51-60	19 (27)	14 (20)
	≥61	4 (6)	3 (4)
**Academic degree, n (%)**			
	None	19 (27)	17 (24)
	Diploma	10 (14)	15 (21)
	Doctorate	10 (14)	4 (6)
	Master of science	0 (0)	2 (3)
	Master of health business	0 (0)	1 (1)
	Other	32 (45)	34 (49)
**Work experience (years), n (%)**			
	≤1	3 (4)	9 (13)
	2-5	12 (17)	15 (21)
	6-10	12 (17)	11 (16)
	≥11	44 (62)	35 (50)
**Previous work experience in different pharmaceutical fields, n (%)**			
	Community pharmacy	70 (99)	62 (89)
	Hospital pharmacy	10 (14)	10 (14)
	Teaching and research	4 (6)	12 (17)
	Pharmaceutical industry	6 (9)	12 (17)
	Other	3 (4)	6 (9)
**Job position in community pharmacy, n (%)**			
	Pharmacy owner	17 (24)	9 (13)
	Branch manager	14 (20)	10 (14)
	Employee	39 (55)	42 (60)
	Other	0 (0)	2 (3)
	No information	1 (1)	7 (10)
**Currently involved in self-medication counseling, n (%)**			
	Yes	65 (92)	62 (89)
	No	5 (7)	1 (1)
	No information	1 (1)	7 (10)

### Primary Outcome Parameter: Quality Score

**Figure 3:**
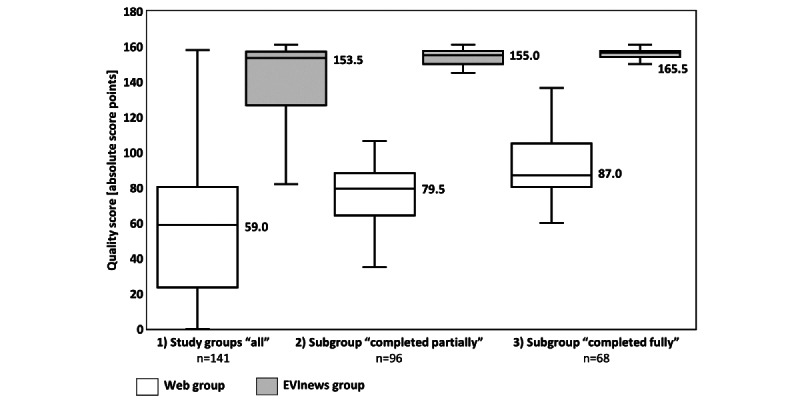
Boxplots of quality scores between study groups and subgroups. The whiskers represent the minimum and the maximum of quality scores. The values represent the medians.

The median quality score of the Web group (n=71 pharmacists) was 32.8% (59.0 out of 180.0 points) and that of the EVInews group (n=70) was 85.3% (153.5 out of 180.0 points, *P*<.001; Figure 3). The variability of the quality score in the Web group (IQR 23.0-80.5) was larger than that in the EVInews group (IQR 125.1-157.0). Table 2 displays all quality score results.

**Table 2:** Differences in quality scores between study groups based on the rating scheme evaluation.

Search task statements^a^	Measures of location and dispersion (quality score points; %), median (n/n; IQR)							
	All study groups			Subgroup “completed partially”			Subgroup “completed fully”	
	Web group (n=71)	EVInews group (n=70)	Web group (n=39)		EVInews group (n=57)	Web group (n=22)		EVInews group (n=46)
Indication 1: female AGA^b^; total quality score	38.9 (35.0/90.0; 0.0-44.0)	90.0 (81.0/90.0; 77.8-83.0)	46.1 (41.5/90.0; 35.0-54.5)		90.0 (81.0/90.0; 80.0-83.0)	53.1 (47.8/90.0; 36.6-57.3)		90.0 (81.0/90.0; 80.0-83.3)
Causes of female AGA	34.5 (10.0/29.0; 0.0-13.0)	89.7 (26.0/29.0; 24.0-27.0)	41.4 (12.0/29.0; 9.0-18.0)		89.7 (26.0/29.0; 24.0-27.0)	44.8 (13.0/29.0; 10.5-18.6)		91.4 (26.5/29.0; 24.0-27.0)
Clinical manifestation of female AGA	37.9 (11.0/29.0; 0.0-15.5)	93.1 (27.0/29.0; 24.0-27.0)	44.8 (13.0/29.0; 11.0-18.0)		93.1 (27.0/29.0; 25.8-27.0)	55.2 (16.0/29.0; 11.9-18.6)		93.1 (27.0/29.0; 26.8-27.0)
Efficacy of topical minoxidil	34.5 (10.0/29.0; 0.0-17.0)	93.1 (27.0/29.0; 25.0-27.0)	48.3 (14.0/29.0; 10.0-18.0)		93.1 (27.0/29.0; 26.0-27.0)	51.0 (14.8/29.0; 10.0-20.0)		93.1 (27.0/29.0; 26.0-27.0)
Indication 2: recurrent herpes labialis; total quality score	30.6 (27.5/90.0; 0.0-38.5)	81.1 (73.0/90.0; 49.8-75.1)	38.9 (35.0/90.0; 27.5-48.5)		81.7 (73.5/90.0; 70.8-76.0)	43.1 (38.8/90.0; 33.4-57.3)		82.2 (74.0/90.0; 73.0-76.0)
Administration of lemon balm leaf extract	31.0 (9.0/29.0; 0.0-16.5)	82.8 (24.0/29.0; 23.4-25.0)	48.3 (14.0/29.0; 8.5-17.0)		82.8 (24.0/29.0; 24.0-25.0)	51.0 (14.8/29.0; 10.5-18.6)		84.5 (24.5/29.0; 24.0-25.0)
Resistance situation of topical antiherpetic drugs	25.9 (7.5/29.0; 0.0-15.5)	86.2 (25.0/29.0; 23.0-25.0)	31.0 (9.0/29.0; 7.5-25.0)		86.2 (25.0/29.0; 25.0-25.0)	37.9 (11.0/29.0; 8.4-25.5)		86.2 (25.0/29.0; 25.0-25.0)
Differences in healing times of combination vs monotherapy (aciclovir and hydrocortisone)	0.0 (0.0/29.0; 0.0-10.5)	77.6 (22.5/29.0; 13.5-24.0)	36.2 (10.5/29.0; 7.5-12.0)		77.6 (22.5/29.0; 21.0-24.0)	37.2 (10.8/29.0; 9.4-14.9)		82.8 (24.0/29.0; 22.4-14.0)
Total quality score (maximum 180 points)	32.8 (59.0/180.0; 23.0-80.5)	85.3 (153.5/180.0; 125.1-157.0)	44.2 (79.5/180.0; 60.5-89.5)		86.1 (155.0/180.0; 148.8-157.5)	48.3 (87.0/180.0; 78.8-111.1)		86.9 (156.5/180.0; 153.8-157.6)

^a^The statements can be found in Table S1 in Multimedia Appendix 1.

^b^AGA: androgenetic alopecia.

### Secondary Outcome Parameters

#### Subgroup Analysis

Results of the subgroup analysis of the subgroups entitled “completed partially” and “completed fully” are shown in Table 2 and illustrated in Figure 3.

#### Quality Scores per Category

Quality scores per rating scheme category were calculated and revealed statistically significant differences, as shown in Table 3.

**Table 3:** Quality scores for the rating scheme categories.

	Rating scheme category	Measures of location and dispersion (quality score points; %), median (n/n; IQR)							
		All study groups			Subgroup “completed partially”			Subgroup “completed fully”	
		Web group (n=71)	EVInews group (n=70)	Web group (n=39)		EVInews group (n=57)	Web group (n=22)		EVInews group (n=46)
Category transparency (maximum 78 points)		25.6 (20.0/78.0; 9.5-30.5)	84.6 (66.0/78.0; 55.0-66.0)	36.5 (28.5/78.0; 20.0-34.0)		84.6 (66.0/78.0; 65.8-66.0)	42.3 (33.0/78.0; 27.9-47.8)		84.6 (66.0/78.0; 66.0-66.0)
Category quality of evidence (maximum 54 points)		14.8 (8.0/54.0; 1.0-15.0)	87.0 (47.0/54.0; 40.0-47.0)	20.4 (11.0/54.0; 6.5-15.0)		74.1 (40.0/54.0; 40.0-40.0)	28.3 (15.3/54.0; 11.4-23.6)		87.0 (47.0/54.0; 47.0-47.0)
Category usability (maximum 6 points)		33.3 (2.0/6.0; 0.0-3.0)	83.3 (5.0/6.0; 0.8-6.0)	33.3 (2.0/6.0; 1.0-2.0)		33.3 (2.0/6.0; 1.0-2.0)	33.3 (2.0/6.0; 1.0-2.0)		33.3 (2.0/6.0; 1.0-2.0)

#### Characterization of Web-Based Information Sources

The web-based information sources that were provided by the participants for the search task varied greatly, as illustrated in Multimedia Appendix 3.

A total of 254 out of potentially 426 web-based information sources (6 statements × 71 participants) were given by 71 participants within the study arm of the web group to verify 6 health-related statements. A list of references was enclosed in 42.9% (109/254) of all web-based sources. Information about the author was provided in 69.7% (177/254) of all the web-based sources, and 34.3% (87/254) were published within the last 3 years (reference date February 1, 2021).

#### Correlational Analysis of Sociodemographic Characteristics and Self-evaluation on Quality Score

The results of the correlational analysis are shown in Table 4. A 2-tailed statistical significance was only detected for the positive correlation between the participant’s estimated annual frequency of searches and quality scores for both study groups and a negative correlation between the age, professional experience, and quality scores within the EVInews group.

**Table 4:** Impact of sociodemographic data as well as self-reflection on skills and quality scores (N=141).

Variables			Web group (n=71)		EVInews group (n=70)
**Quality score and participant’s age**					
	Correlation coefficient *r_s_*	−0.187		−0.360	
	*P* value (2-tailed)	.12		.002	
**Quality score and professional experience** **(years)**					
	Correlation coefficient *r_s_*	−0.205		−0.268	
	*P* value (2-tailed)	.09		.03	
**Quality score and academic degree**					
	Correlation coefficient *r_s_*	0.073		0.130	
	*P* value (2-tailed)	.55		.28	
**Quality score and estimated frequency of searches with literature databases per year**					
	Correlation coefficient *r_s_*	0.387		0.274	
	*P* value (2-tailed)	.009		.04	
**Quality score and self-reflection on ability to search for evidence related to OTC^a^ products**					
	Correlation coefficient *r_s_*	0.033		0.147	
	*P* value (2-tailed)	.83		.27	

^a^OTC: over-the-counter.

#### Self-evaluation of Search Skills and Feedback

The responses of the self-evaluation (web group, n=44; EVInews group, n=58) were as follows. A total of 25% (11/44) of participants within the web group agreed fully that they were able to find the desired information in contrast to 71% (41/58) of the EVInews group. Furthermore, 46% (20/44) of the participants within the web group indicated that they conducted between no literature searches with literature databases such as MEDLINE via PubMed and 39% (17/44) to up to 12 per year in comparison with 40% (23/58) of the EVInews database users and 35% (20/58), respectively. Moreover, 39% (17/44) of participants of the web group estimated their search ability to find evidence-based self-medication information to be “rather good” and 5% (2/44) to be “very good.” Within the EVInews group, 59% (34/58) regarded their search ability to be “rather good” and 9% (5/58) to be “very good.” A total of 72% (42/58) of the EVInews group indicated that they were not familiar with the database before the study participation.

#### Time Needed to Perform the Search Task

The times needed for the search task completion are shown in Table 5.

The median for the search task completion was 25.4 (IQR 12.7-43.5) minutes in the web group and 19.7 (IQR 13.6-28.4) minutes in the EVInews group. The completion times include participants who chose the escape option “I don’t know.”

**Table 5:** Time needed to perform the search task

	Study groups “all” (*P*=.12)		Subgroup “completed partially” (*P*=.04)		Subgroup “completed fully” (*P*=.14)	
	Web group (n=71)	EVInews group (n=70)	Web group (n=43)	EVInews group (n=59)	Web group (n=22)	EVInews group (n=46)
Time needed for search-task completion (minutes), median (IQR)	25.4 (12.7-43.5)	19.7 (13.6-28.4)	28.5 (18.0-40.0)	21.4 (17.2-28.6)	28.7 (17.7-40.0)	22.4 (28.7-17.7)

### Quality Assurance: Rater Performance

The extent of agreement between the 2 raters measured using the Cohen κ statistic was “almost perfect” (κ=0.81-1) for the rating scheme items 1, 2, 4a, 4d-13, and 15-18. The extent of agreement on items 14 complied with “substantial agreement” (κ=0.780), items 3 and 4c with “moderate agreement” (κ=0.41-0.60), and item 4b with “slight agreement” (κ=0.077). All values were significantly different from zero (*P*<.001). The extent of agreement in the form of an overall interrater descriptor for all rating scheme items accounted to κ=0.854.

## Discussion

### Principal Findings

We found that the median quality score was significantly lower in the web group than in the EVInews group. Additionally, the variability of median quality scores in the web group was significantly larger and less participants completed the search task fully compared to the EVInews group. Web-based sources often did not meet standard quality requirements. The time needed for the search task completion did not reveal any considerable differences between the 2 groups. The data suggest that community pharmacists struggled to find web-based high-quality, evidence-based information sources for self-medication under everyday practice conditions.

### Differences in Quality Scores and Potential Causes

The web group obtained a significantly lower quality score with 32.8% of criteria fulfilled in comparison to 85.3% in the EVInews group. Several explanations for this result are possible. The statistically significant difference in quality scores may be explained by a lack of transparency information of many web-based sources, which ultimately resulted in lower scores. Examples of missing transparency information include publication dates, literature references, or authorship information. An analysis of the categories transparency and quality of evidence confirmed the persisting difference in scores in favor of the EVInews group.

Furthermore, a subgroup analysis was conducted because the difference in quality could be caused by participants who mostly chose the escape answer option “I don’t know.” A significant difference in quality scores remained when only data from participants, who verified all 6 health-related statements, were included.

The differences in quality scores between the participants could also be explained by personal factors such as different levels of work experience, knowledge, and the skills needed to quickly find suitable external evidence on the internet. However, significant differences in sociodemographic data between both groups were not existent. According to our correlational analysis, there was a positive association between the participant’s estimated annual literature searches and quality scores in both study groups. A negative correlation between the participant’s age as well as professional experience in years and quality scores was only observed within the EVInews group. This may indicate that with increasing practical literature search experience, the ability to find appropriate evidence could increase but with increasing age and professional experience decrease. The factors age and professional experience however only seemed to be associated with the quality scores of the EVInews group. It should be taken into consideration that EbPharm has been established in recent years, and therefore pharmacists who were trained over 30 years ago may not be familiar with this concept. Abu Farha et al [10] similarly demonstrated a negative correlation among pharmacists between the familiarity with evidence-based medicine terms and professional experience.

### Need for Customized Evidence-Based Information Sources for Pharmacists

Most of the web-based information resources consisted of tertiary literature in the form of nonscientific pharmacy journals of varying quality and timeliness. Some information resources were 20 years old. Despite its simple nature as a document-oriented database, the majority of participants using the EVInews database “agreed fully” (41/58, 71%) in contrast to the web group (11/44, 25%) that they were able to find the desired information. Users need to sign in before accessing the EVInews database. The login with a username and password is comparable to other information platforms of this kind. This may be regarded as an obstacle to the database’s usage for everyday practice. However, when customers or other health professionals seek evidence-based advice related to OTC products, pharmacists may often not be able to immediately provide all the necessary information anyways. These cases require a search for external evidence under everyday conditions such as simulated in our study. The ability to find the desired information when confronted with such a search task is therefore of great importance and even greater importance than retrieval speed from our point of view. Nonetheless, it remains unknown how much low-quality sources impact the overall consultation quality. Furthermore, the dropout rate was greater in the web group. Only 22 participants within the web group in comparison with 46 within the EVInews group completed the entire search task and verified all 6 health-related statements.

Although the information retrieval times did not differ significantly, the participant’s search results under everyday practice conditions clearly did. An implication of this is the possibility that the participants were simply overwhelmed with the search task because they lacked the necessary search skills and therefore did not complete it fully. Subsequently, this leads to the question: what information do pharmacists rely on when selling OTC products if they cannot find it? These data may furthermore suggest that there is a need for training on how to find and appraise evidence as well as evidence-based information sources for community pharmacists. Because a lack of information sources and search skills is a common barrier to the implementation of EbPharm, information tools may help reduce the evidence-to-practice gap. The EVInews database fulfilled a majority of the standard quality requirements. Once further adjusted, it may be a promising electronic information tool for community pharmacists for evidence-based counseling.

### Limitations

A Hawthorne effect may have influenced the participants’ search behavior in both groups, prompting them to look for the very best evidence in the trial setting. Because of the web-based format, they were, however, not directly observed, and therefore this effect probably plays a minor role.

Raters and participants were not blinded. Because the type of information sources the users were allowed to use was predefined, blinding was not possible. To minimize subjective evaluation, a quantitative approach for the quality evaluation with objective criteria was chosen to ascertain a robust rating performance.

Only 2 OTC-relevant indications were chosen for the search task (Table S1 in Multimedia Appendix 1). It remains questionable whether these 2 indications were adequate for the search task. Customers’ inquiries regarding the evidence or verification of advertising statements for the treatment of female androgenetic alopecia or recurrent herpes labialis are nonetheless common in everyday counseling practice and were therefore included in this study. Furthermore, on the basis of the pretests and pilot tests, we made sure that there is enough citable literature in German and English available to complete the search task.

The exclusion of tertiary print literature was due to technical difficulties when conducting the web-based survey. However, electronic information sources are frequently used among pharmacists nowadays, especially for the verification of health-related claims.

The rating scheme was not validated before the survey was conducted; hence extensive pretests and pilot tests were performed.

### Conclusions

Community pharmacists struggled to find high-quality electronic self-medication–related information sources under everyday practice conditions in the web group. This is of great importance as self-medication recommendations or simply the verification of statements should not be based on information resources of questionable quality. The search results of the EVInews group met many of the predefined standard quality criteria. Taken together, the findings suggest that there is a need for customized information sources for pharmacists. Evidence-based information tools such as the EVInews database may help in reducing the evidence-to-practice gap.

## Appendix

Multimedia Appendix 1 Search-task health-related statements.

Multimedia Appendix 2 Rating Scheme.

Multimedia Appendix 3 Type and frequency distribution of web-based information sources.

Multimedia Appendix 4 CONSORT-eHEALTH checklist (V 1.6.1).

## Abbreviations

ABDA: Federal Union of German Associations of Pharmacists
EbPharm: evidence-based pharmacy
OTC: over-the-counter
