# Supplementary material for: Enhancing Evidence-Based Pharmacy by Comparing the Quality of Web-Based Information Sources to the EVInews Database: Randomized Controlled Trial With German Community Pharmacists
Source: J Med Internet Res. 2023 Jun 21;25:e45582. doi: 10.2196/45582 (PMC10337305; doi:10.2196/45582)
Supplement: Multimedia Appendix 1 [file jmir_v25i1e45582_app1.doc]

## Multimedia Appendix

Supplement 1.

Search-task health-related statements

| ***Female androgenetic Alopecia*** – original (translated) statements | |
| --- | --- |
| 1 | Hormonal regulation disorders are often the cause of androgenetic alopecia in women. |
| 2 | In the female form of androgenetic alopecia, there is often a thinning of the hair starting from the center of the head towards the rest of the head. |
| 3 | There is evidence (proof) for the efficacy of topical minoxidil in androgenetic alopecia in women. |
| ***Recurrent Herpes labialis*** – original (translated) statements | |
| 1 | Topical lemon balm leaf extract for recurrent cold sores should be applied at the onset of the first prodromes. |
| 2 | In contrast to nucleoside analogues (aciclovir, pencivir), herpes simplex viruses are not yet known to be resistant to topical lemon balm leaf extract. |
| 3 | A significant improvement in cold sore time until healing has not been demonstrated with topical combination therapy (aciclovir and hydrocortisone) compared to topical monotherapy (aciclovir). |
