# Supplementary material for: Enhancing Evidence-Based Pharmacy by Comparing the Quality of Web-Based Information Sources to the EVInews Database: Randomized Controlled Trial With German Community Pharmacists
Source: J Med Internet Res. 2023 Jun 21;25:e45582. doi: 10.2196/45582 (PMC10337305; doi:10.2196/45582)
Supplement: Multimedia Appendix 2 [file jmir_v25i1e45582_app2.docx]

## Multimedia Appendix

Supplement 2.

Rating Scheme

| **Rating Categories** | **Main rating criteria** | Rating criterion number | Rating Questions | Point Allocation | Notes |
| --- | --- | --- | --- | --- | --- |
| **Accuracy** | Source disclosure | 1 | Was a source provided at all? | No: 0 points | No source inserted in the text field  In case no source was provided, no further quality score evaluation will follow. |
|  |  |  |  | Yes: 1 point | A source is inserted in the text field. |
|  | Quote disclosure | 2 | Was a quote provided at all? | No: 0 points | No quote inserted in the text field. |
|  |  |  |  | Yes: 1 point | A quote is inserted in the text field. |
|  | Source adequacy | 3 | Quote can be found in the provided source? | No: skip the following questions and go to Rating Criterion 5, if source seems adequate*. If source does not appear to be suitable regarding the content, the quality analysis of this statement will terminated at this point. | Quote will be searched within the provided source by the search function (keyboard shortcut Strg+F on Windows and Linux)  * We define an adequate source as an information source that addresses one of the following topics:  Indication Herpes labialis  A) Lemon balm extract (for treatment of Herpes labialis)  B) Antiherpetic drugs  C) Nucleoside mono- and combination therapy for Herpes labialis  Indication Female adrogenetic Alopecia  A) Female androgenetic alopecia  B) Female Androgenetic alopecia symptoms  C) Topical minoxidil for (female) androgenetic alopecia |
|  |  |  |  | Yes: 1 point | Quote was not found word-for-word, but the gist of the verifying statement was provided |
|  |  |  |  | Yes: 2 points | Quote was found word-for-word |
|  | Agreed Procedure:   - No source and quote provided: termination of quality analysis of the statement – equivalent to 0 points - If no source is provided: equivalent to 0 points.   For the quality analysis, a source must be provided. If a quote was entered without a source, it cannot be considered for the quality evaluation and therefore will result in 0 points. The source field can only be left blank when the item option: “I don’t know” was selected.   - If no quote is provided:   The quality score of the source will be analyzed solely (rating category: Transparency, Quality of Evidence & Usability, Criterion Number 5 – 18, Max. 22 points out of 29 points). The rating category “Accuracy” will not be taken into account and result therefore in 0 points.   - If an inaccessible source is provided (e.g. pdf):   Attempts will be made to access the provided source. After the study termination in May, options to access these sources will be discussed during an expert panel. In case all attempts fail, a quality score cannot be analyzed and the concerned item will result in 0 points.   - If source- and text field are not inserted in the designated text fields:   Scenario 1 – within the same statement response items: If the source and quote were inserted into the wrong text fields within one response item, the quote and source will still be taken into consideration for the quality score and allocated to the correct text fields.  Scenario 2– within different statement response items:  When quotes and sources were inserted in the wrong text fields, but seem adequate to verify the statements and were only placed in the wrong order (eg. response items a-c for each statement and indication), a full quality analysis will be conducted and the quotes and/or sources will be designated to the correct text fields.  Scenario 3 - a non-matching quote or source was provided (quote was not found within the source provided or did not align with the selected response)   - When source and quote were inserted ≥twice and/or ≥ 1 quote did not refer to the proposed statement content twice, only one full quality analysis will be undertaken for the correct quote. The other quotes and sources will not be taken into account for the quality analysis. - In case the selected response item (I agree, I don’t agree) does not align with the content of the provided quote, the quality analysis will be performed solely for the Rating Category: Transparency, Quality of Evidence & Usability (Criterion Number 5 – 18, Max. 22 points out of 29 points). - When source and quote were inserted ≥twice for one of the following 2 indications: Herpes labialis, androgenetic female alopecia, only one quote will be taken into consideration for the full quality analysis. For the other 2 statements only the quality source evaluation will be conducted, in case the source is considered “adequate”. When a source is rated as “inadequate”, a final discussion within an expert panel will be held. | | | | |
|  | Validity | 4 | Sub-Category a:  Did the participant choose the right answer (“Ich stimme Aussage XY zu”) to verify the statement? | Yes: move on to Rating Criterion 4b |  |
|  |  |  |  | No: skip to Rating Criterion 5. |  |
|  |  |  | Was the quote’s content proving the statement?  Sub-Category b:  Quote refers to the content of the statement?  The statement’s main theme is addressed in the quote? | Yes: 1 point  Continue with 4c | Statements:  Indication Herpes labialis  A) (early) Recommended application (time) of topical lemon balm extract (for treatment of Herpes labialis)  B) Resistance situation of antiherpetic drugs (Nucleosides vs. topical lemon balm extract)  C) Differences in healing times between the nucleoside mono- and combination therapy  Indication Female adrogenetic Alopecia  A) Causes of female androgenetic alopecia  B) (frontal) “hair thinning“ as a female androgenetic alopecia symptom  C) Efficacy proof for (female) androgenetic alopecia/ efficacy proof for topical minoxidil |
|  |  |  |  | No: 0 points  In case No was selected, no further validity analysis will be performed. Skip to Rating Criterion 5. |  |
|  |  |  | Was the quote’s content proving the statement?  Sub-Category c:  Is the quote suitable for verifying the statement? *(logically,-rationally comprehensible)* | No | In case No was selected, no further validity analysis will be conducted. Skip to Rating Criterion no. 5. |
|  |  |  |  | Yes | Continue with 4c |
|  |  |  | Was the quote’s content proving the statement?  Sub-Category d:  Quote provides proof to confirm or disprove the statement? | Summative Content analysis will be conducted. Identification of relevant keywords from at least 2 a priori defined categories are included within quote.  The quote should contain at least one of the predefined key words. A List of key words is provided within the Excel sheet used for the data analysis. | |
|  |  |  |  | Partially yes:  1 point | The quote did contain at least one key word from 1 category. |
|  |  |  |  | Yes: 2 points | The quote did contain at least one key word from 2 categories. |
| **Transparency** | Authorship disclosure | 5 | Information about author(s) disclosed at all? | No: 0 points | No author name given |
|  |  |  |  | Yes: 1 point | Author(s) name(s) given |
|  |  | 6 | Was more than author involved in composing the source?  *(especially in case several authors are listed)* | No: 0 points | Only one author name was provided |
|  |  |  |  | Yes: 1 point | ≥ 1 author composed the information source and names were provided |
|  |  | 7 | Information about affiliation(s) disclosed? | No: 0 points | Within source or medium’s website no information about author’s affiliation can be found |
|  |  |  |  | Yes: 1 point | information about author’s affiliation can be found |
|  |  | 8 | Was/Were the author(s) qualified enough to compose information? | No: 0 points | No information about the author’s qualification can be found within the source.  We define qualified authors for OTC- counseling health information as Health Care Professionals from one of the following or related fields: Pharmacy, Medicine, Nursing, (Chemistry, Biology or related fields for symptom based statement or resistance statement) or provided affiliation indicates that knowledge for composing the information was sufficient. |
|  |  |  |  | Yes: 1 point | Information about the author’s qualification can be found within the source. |
|  | Timeliness/Currency | 9 | Date of publication or update disclosed? | No: 0 points | No publication date is disclosed. |
|  |  |  |  | Yes: 1 point | Publication date is disclosed. |
|  |  | 10 | Was the date of creation within the past 3 years? | No: 0 points | Reference date: initiation of study (01.02.2021) |
|  |  |  |  | Yes: 1 point | Reference date: initiation of study (01.02.2021) |
|  | Information editorial review process | 11 | Is it possible to contact the authors or an editorial board for further information? | No: 0 points | No option to contact authors such as an E-Mail address, phone number or query box can be found. |
|  |  |  |  | Partially Yes: 1 point | Author’s /Editorial board’s contact information are displayed on the article/website |
|  |  |  |  | Yes: 2 points | Author’s /Editorial board’s contact information are displayed on the information source |
|  |  | 12 | Is the information source a reviewed publication? | No: 0 points | No information about peer review/revision system/correction disclosed on article or website. |
|  |  |  |  | Yes: 1 point | Publication/medium undergoes review process |
|  |  |  |  | Yes: 2 points | Publication/medium undergoes peer review process |
|  | Conflict of interests/bias | 13 | Are conflicts of interests labelled? | No: 0 points | No information regarding conflicts of interests/sources of bias can be found within the source or website |
|  |  |  |  | Yes: 1 point | A statement regarding conflicts of interests/sources of bias should be included within information source or website |
|  |  | 14 | Is advertisement included within the information source? | No: 1 point | No advertisement was included within the source |
|  |  |  |  | Yes: 0 points | Advertisement is included |
|  | Literature sources | 15 | Are literature sources provided? | No: 0 points | No literature sources within a bibliography are listed within the source, literature source must be accessible/identifiable through provided information |
|  |  |  |  | Yes: 1 point | Literature sources within a bibliography are listed within the source, literature source must be accessible/identifiable through provided information |
| **Quality of evidence** | Quality of evidence | 16 | Does the source provide any information about the quality of evidence? | No: 0 points | No Information on evidence levels/grades/quality of main information source (according to e.g. CEBM, medical guidelines) is mentioned within the entire information source. Information will be found through searching for specific key words (e.g. evidence). |
|  |  |  |  | Partially yes: 1 point | Information source mentions that information about evidence level, grade, quality reference is existent for matter of interest, but does not specify |
|  |  |  |  | Yes: 2 points | Information source specifies evidence level/grade/ reference quality and or reporting quality or risk of bias (eg. GRADE, CONSORT, AMSTAR 1&2 , CASP) |
|  |  | 17 | Is the source based on reliable, evidence-based data?  (at least one type of evidence listed within the right column is included within the list of references)  (Exception: in case of an expert opinion, no literature source quoting the expert needs to be provided) | Systematic reviews/meta-analysis: 7 points | |
|  |  |  |  | Medical guidelines: 6 points | |
|  |  |  |  | RCTs: 5 points | |
|  |  |  |  | Cohort studies: 4 points | |
|  |  |  |  | Case control studies: 3 points | |
|  |  |  |  | In-vitro / in-vivo: 2 points | |
|  |  |  |  | Expert opinion/background opinion/tertiary literature: 1 point | |
|  |  |  |  | No information disclosed: 0 points | |
| Preliminary quality score | | | | 29 | |
| usability | Time needed to perform the search-task | 18 | Was the information access per case vignette (3 statements) possible within 15 minutes (900 seconds)? | No: 0 points | Indication 1 or 2: time until completion did exceed 15 minutes |
|  |  |  |  | 1 point | Indication 1 or 2: time until completion did not exceed 15 minutes in case 1 statement was verified |
|  |  |  |  | 2 points | Indication 1 or 2: time until completion did not exceed 15 minutes in case 2 statements were verified |
|  |  |  |  | Yes: 3 points | Indication 1 or 2: time until completion did not exceed 15 minutes in case all 3 statements were verified |
| Quality score per statement | | | | Maximum 29 points, minimum: 0 points | |
| Quality score per case vignette (3 statements) | | | | Maximum 90 points, minimum: 0 points | |
| Quality score (6 statements) | | | | Maximum 180 points, minimum: 0 points | |
