# Supplementary figures and images for: Enhancing Evidence-Based Pharmacy by Comparing the Quality of Web-Based Information Sources to the EVInews Database: Randomized Controlled Trial With German Community Pharmacists
Source: J Med Internet Res. 2023 Jun 21;25:e45582. doi: 10.2196/45582 (PMC10337305; doi:10.2196/45582)

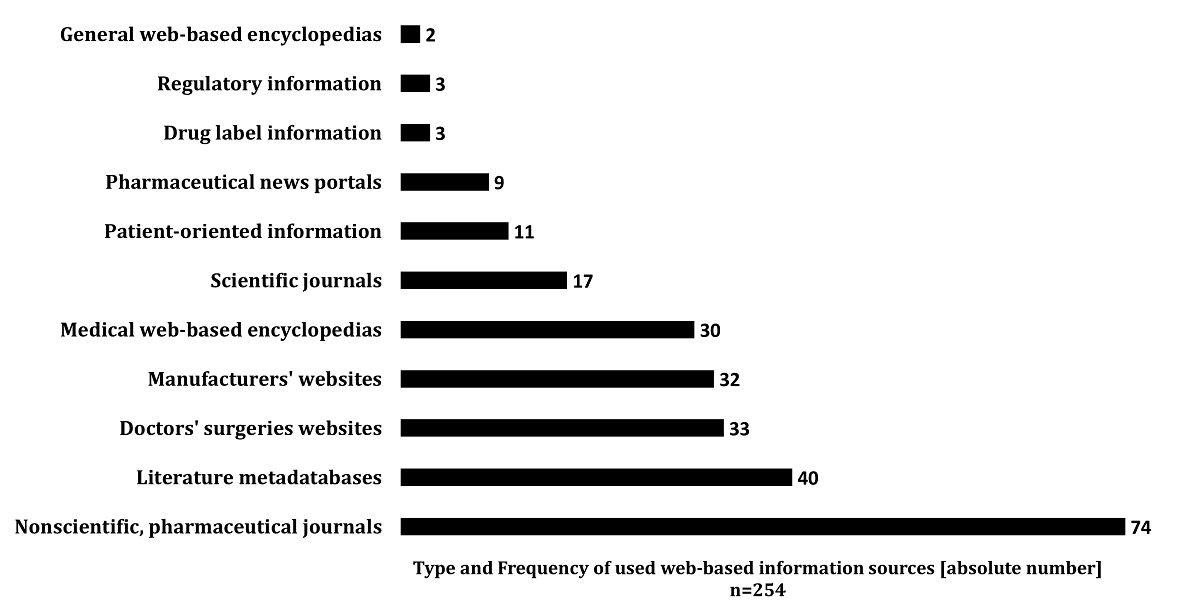

Supplement: Multimedia Appendix 3 [file jmir_v25i1e45582_app3.png]
