# Supplementary material for: Enhancing Evidence-Based Pharmacy by Comparing the Quality of Web-Based Information Sources to the EVInews Database: Randomized Controlled Trial With German Community Pharmacists
Source: J Med Internet Res. 2023 Jun 21;25:e45582. doi: 10.2196/45582 (PMC10337305; doi:10.2196/45582)
Supplement: Multimedia Appendix 4 [file jmir_v25i1e45582_app4.pdf]

CONSORT-EHEALTH (V 1.6.1) - Submission/Publication Form

The CONSORT-EHEALTH checklist is intended for authors of randomized trials evaluating web-based and Internet-based applications/interventions, including mobile interventions, electronic games (incl multiplayer games), social media, certain telehealth applications, and other interactive and/or networked electronic applications. Some of the items (e.g. all subitems under item 5 - description of the intervention) may also be applicable for other study designs.

The goal of the CONSORT EHEALTH checklist and guideline is to be
a) a guide for reporting for authors of RCTs,
b) to form a basis for appraisal of an ehealth trial (in terms of validity)

CONSORT-EHEALTH items/subitems are MANDATORY reporting items for studies published in the Journal of Medical Internet Research and other journals / scientific societies endorsing the checklist.

Items numbered 1., 2., 3., 4a., 4b etc are original CONSORT or CONSORT-NPT (non- pharmacologic treatment) items.
Items with Roman numerals (i., ii, iii, iv etc.) are CONSORT-EHEALTH extensions/clarifications.

As the CONSORT-EHEALTH checklist is still considered in a formative stage, we would ask that you also RATE ON A SCALE OF 1-5 how important/useful you feel each item is FOR THE PURPOSE OF THE CHECKLIST and reporting guideline (optional).

Mandatory reporting items are marked with a red *.
In the textboxes, either copy & paste the relevant sections from your manuscript into this form - please include any quotes from your manuscript in QUOTATION MARKS,
or answer directly by providing additional information not in the manuscript, or elaborating on why the item was not relevant for this study.

YOUR ANSWERS WILL BE PUBLISHED AS A SUPPLEMENTARY FILE TO YOUR PUBLICATION IN JMIR AND ARE CONSIDERED PART OF YOUR PUBLICATION (IF ACCEPTED).
Please fill in these questions diligently. Information will not be copyedited, so please use proper spelling and grammar, use correct capitalization, and avoid abbreviations.

DO NOT FORGET TO SAVE AS PDF _AND_ CLICK THE SUBMIT BUTTON SO YOUR ANSWERS ARE IN OUR DATABASE !!!

Citation Suggestion (if you append the pdf as Appendix we suggest to cite this paper in the caption):
Eysenbach G, CONSORT-EHEALTH Group
CONSORT-EHEALTH: Improving and Standardizing Evaluation Reports of Web-based and Mobile Health Interventions
J Med Internet Res 2011;13(4):e126

URL: http://www.jmir.org/2011/4/e126/
doi: 10.2196/jmir.1923
PMID: 22209829

In Google anmelden, um den Fortschritt zu speichern. Weitere Informationen

* Erforderlich

Your name
*

Jennifer Maria Alexa, Thilo Bertsche

Primary Affliation (short), City, Country *

Leipzig University, Leipzig

Your e-mail address
*

jennifer.alexa@uni-leipzig.de,
thilo.bertsche@uni-leipzig.de

Title of your manuscript *

Provide the (draft) title of your manuscript.

Enhancing evidence-based Pharmacy: The quality of web-based information sources compared to the EVInews-database - A randomized controlled trial with German community pharmacists

Name of your App/Software/Intervention *

If there is a short and a long/alternate name, write the short name first and add the long name in brackets.

web-based information sources,
control: EVInews-database

Evaluated Version (if any)

e.g. "V1", "Release 2017-03-01", "Version

not applicable

Language(s) *

What language is the intervention/app in? If multiple languages are available, separate by comma (e.g. "English, French")

German

URL of your Intervention Website or App

e.g. a direct link to the mobile app on app in appstore (itunes, Google Play), or URL of the website. If the intervention is a DVD or hardware, you can also link to an Amazon page.

Not applicable. Control: EVInews database: https://www.evinews.de/index.php?id=login

URL of an image/screenshot (optional)

Primary Medical Indication/Disease/Condition *

e.g. "Stress", "Diabetes", or define the target group in brackets after the condition, e.g. "Autism (Parents of children with)", "Alzheimers (Informal Caregivers of)"

Not applicable. This intervention targeted community pharmacists' electronic information sources for evidence-based self-medication counseling.

Primary Outcomes measured in trial *

comma-separated list of primary outcomes reported in the trial

The primary outcome parameter was the difference in quality scores between the study groups.

Secondary/other outcomes

Are there any other outcomes the intervention is expected to affect?

Secondary outcome parameters include a subgroup analysis, quality scores of the rating scheme categories transparency, quality of evidence and usability, the characterization of web-based information sources, a correlational analysis of sociodemographic characteristics and quality scores, a self-evaluation of search skills by the participants and assessment of the time needed to perform the search-task.

Accessibility *

Can an enduser access the intervention

- (x) access is free and open
- ( ) access only for special usergroups, not open
- ( ) access is open to everyone, but requires payment/subscription/in-app purchases
- ( ) app/intervention no longer accessible
- ( ) Sonstiges:

Participants used web-based information sources of their choice, which were accessible. EVInews-database is free of charge and accessible after registration. Trial participants in the EVInews-group were given a test account to use the database.

Recommended "Dose" *

What do the instructions for users say on how often the app should be used?

- ( ) Approximately Daily
- ( ) Approximately Weekly
- ( ) Approximately Monthly
- ( ) Approximately Yearly
- (x) "as needed"
- ( ) Sonstiges:

Approx. Percentage of Users (starters) still using the app as recommended after 3 months *

- ( ) unknown / not evaluated
- ( ) 0-10%
- ( ) 11-20%
- ( ) 21-30%
- ( ) 31-40%
- ( ) 41-50%
- ( ) 51-60%
- ( ) 61-70%
- ( ) 71%-80%
- ( ) 81-90%
- ( ) 91-100%
- ( ) Sonstiges:
- (x) Not applicable. Participants participated anonymously. They either used web-based sources of their choice or the EVInews-database through a test-account. The test account was only available throughout the duration of the trial.

Article Preparation Status/Stage *

At which stage in your article preparation are you currently (at the time you fill in this form)

- ( ) not submitted yet - in early draft status
- (x) not submitted yet - in late draft status, just before submission
- ( ) submitted to a journal but not reviewed yet
- ( ) submitted to a journal and after receiving initial reviewer comments
- ( ) submitted to a journal and accepted, but not published yet
- ( ) published
- ( ) Sonstiges:

Overall, was the app/intervention effective? *

- ( ) yes: all primary outcomes were significantly better in intervention group vs control
- ( ) partly: SOME primary outcomes were significantly better in intervention group vs control
- ( ) no statistically significant difference between control and intervention
- ( ) potentially harmful: control was significantly better than intervention in one or more outcomes
- ( ) inconclusive: more research is needed
- (x) Sonstiges:

All primary outcomes were significantly better in the control group (EVInews-group) compared to the intervention group (web-based group). Therefore, the control appears to be more effective.

Journal *

If you already know where you will submit this paper (or if it is already submitted), please provide the journal name (if it is not JMIR, provide the journal name under "other")

- ○ not submitted yet / unclear where I will submit this
- ● Journal of Medical Internet Research (JMIR)
- ○ JMIR mHealth and UHealth
- ○ JMIR Serious
- ○ Games JMIR Mental
- ○ Health JMIR Public
- ○ Health
- ○ JMIR Formative Research
- ○ Other JMIR sister journal
- ○ Sonstiges:

Is this a full powered effectiveness trial or a pilot/feasibility trial? *

- ○ Pilot/feasibility
- ● Fully powered

Manuscript tracking number *

If this is a JMIR submission, please provide the manuscript tracking number under "other" (The ms tracking number can be found in the submission acknowledgement email, or when you login as author in JMIR. If the paper is already published in JMIR, then the ms tracking number is the four-digit number at the end of the DOI, to be found at the bottom of each published article in JMIR)

- ● no ms number (yet) / not (yet) submitted to / published in JMIR
- ○ Sonstiges:

TITLE AND ABSTRACT

1a) TITLE: Identification as a randomized trial in the title

1a) Does your paper address CONSORT item 1a? *

I.e does the title contain the phrase "Randomized Controlled Trial"? (if not, explain the reason under "other")

- (x) yes
- ( ) Sonstiges:

1a-i) Identify the mode of delivery in the title

Identify the mode of delivery. Preferably use “web-based” and/or “mobile” and/or “electronic game” in the title. Avoid ambiguous terms like “online”, “virtual”, “interactive”. Use “Internet-based” only if Intervention includes non-web-based Internet components (e.g. email), use “computer-based” or “electronic” only if offline products are used. Use “virtual” only in the context of “virtual reality” (3-D worlds). Use “online” only in the context of “online support groups”. Complement or substitute product names with broader terms for the class of products (such as “mobile” or “smart phone” instead of “iphone”), especially if the application runs on different platforms.

subitem not at all important

- 1 ( )
- 2 ( )
- 3 ( )
- 4 (x)
- 5 ( )

essential

Does your paper address subitem 1a-i? *

Copy and paste relevant sections from manuscript title (include quotes in quotation marks "like this" to indicate direct quotes from your manuscript), or elaborate on this item by providing additional information not in the ms, or briefly explain why the item is not applicable/relevant for your study

“Enhancing evidence-based Pharmacy: The quality of web-based information sources compared to the EVInews-database - A randomized controlled trial with German community pharmacists”

1a-ii) Non-web-based components or important co-interventions in title

Mention non-web-based components or important co-interventions in title, if any (e.g., “with telephone support”).

subitem not at all important

- 1 ○
- 2 ○
- 3 ○
- 4 ●
- 5 ○

essential

Does your paper address subitem 1a-ii?

Copy and paste relevant sections from manuscript title (include quotes in quotation marks "like this" to indicate direct quotes from your manuscript), or elaborate on this item by providing additional information not in the ms, or briefly explain why the item is not applicable/relevant for your study

Both information tools, consisting of the web-based information sources of choice and the EVInews-database are mentioned within the title. Therefore, no co-interventions or non-web-based components are listed within the title.

1a-iii) Primary condition or target group in the title

Mention primary condition or target group in the title, if any (e.g., “for children with Type I Diabetes”) Example: A Web-based and Mobile Intervention with Telephone Support for Children with Type I Diabetes: Randomized Controlled Trial

subitem not at all important

1 ○

2 ○

3 ○

4 ●

5 ○

essential

Does your paper address subitem 1a-iii? *

Copy and paste relevant sections from manuscript title (include quotes in quotation marks "like this" to indicate direct quotes from your manuscript), or elaborate on this item by providing additional information not in the ms, or briefly explain why the item is not applicable/relevant for your study

“[…] A randomized controlled trial with German community pharmacists”

1b) ABSTRACT: Structured summary of trial design, methods, results, and conclusions

NPT extension: Description of experimental treatment, comparator, care providers, centers, and blinding status.

1b-i) Key features/functionalities/components of the intervention and comparator in the METHODS section of the ABSTRACT

Mention key features/functionalities/components of the intervention and comparator in the abstract. If possible, also mention theories and principles used for designing the site. Keep in mind the needs of systematic reviewers and indexers by including important synonyms. (Note: Only report in the abstract what the main paper is reporting. If this information is missing from the main body of text, consider adding it)

subitem not at all important

1 ○

2 ○

3 ○

4 ○

5 ●

essential

Does your paper address subitem 1b-i? *

Copy and paste relevant sections from the manuscript abstract (include quotes in quotation marks "like this" to indicate direct quotes from your manuscript), or elaborate on this item by providing additional information not in the ms, or briefly explain why the item is not applicable/relevant for your study

“After the approval of the ethics protocol, we performed a quantitative online survey with a search-task as a prospective, randomized, controlled unblinded study. For the search-task, participants were instructed to search for evidence-based information to verify 6 health-related statements from 2 typical self-medication indications. Pharmacists across Germany were invited to participate by E-Mail. After written informed consent, they were automatically, randomly assigned to either use web-based information sources of their choice without the EVInews-database (Web-group) or exclusively the EVInews-database (EVInews-group). The quality of the information sources that were utilized for the search-task was then assessed by two evaluators using a quality score ranging from 100% (180 points, all predefined criteria fulfilled) to 0% (0 points, none of the predefined criteria fulfilled). In case of assessment discrepancies, an expert panel consisting of 4 pharmacists was consulted”.

1b-ii) Level of human involvement in the METHODS section of the ABSTRACT

Clarify the level of human involvement in the abstract, e.g., use phrases like “fully automated” vs. “therapist/nurse/care provider/physician-assisted” (mention number and expertise of providers involved, if any). (Note: Only report in the abstract what the main paper is reporting. If this information is missing from the main body of text, consider adding it)

subitem not at all important

1 ○

2 ○

3 ○

4 ●

5 ○

essential

Does your paper address subitem 1b-ii?

Copy and paste relevant sections from the manuscript abstract (include quotes in quotation marks "like this" to indicate direct quotes from your manuscript), or elaborate on this item by providing additional information not in the ms, or briefly explain why the item is not applicable/relevant for your study

“After the approval of the ethics protocol, we performed a quantitative online survey with a search-task as a prospective, randomized, controlled unblinded study. Pharmacists across Germany were invited to participate by E-Mail. After written informed consent, they were automatically, randomly assigned to either use web-based information sources of their choice without the EVInews-database (Web-group) or exclusively the EVInews-database (EVInews-group). The quality of the information sources that were utilized for the search-task was then assessed by two evaluators […].” The evaluators had no interaction at any point with the users of web-based sources or users of the EVInews-database.

1b-iii) Open vs. closed, web-based (self-assessment) vs. face-to-face assessments in the METHODS section of the ABSTRACT

Mention how participants were recruited (online vs. offline), e.g., from an open access website or from a clinic or a closed online user group (closed usergroup trial), and clarify if this was a purely web-based trial, or there were face-to-face components (as part of the intervention or for assessment). Clearly say if outcomes were self-assessed through questionnaires (as common in web-based trials). Note: In traditional offline trials, an open trial (open-label trial) is a type of clinical trial in which both the researchers and participants know which treatment is being administered. To avoid confusion, use “blinded” or “unblinded” to indicated the level of blinding instead of “open”, as “open” in web-based trials usually refers to “open access” (i.e. participants can self-enrol). (Note: Only report in the abstract what the main paper is reporting. If this information is missing from the main body of text, consider adding it)

subitem not at all important

1 ○

2 ○

3 ○

4 ○

5 ●

essential

Does your paper address subitem 1b-iii?

Copy and paste relevant sections from the manuscript abstract (include quotes in quotation marks "like this" to indicate direct quotes from your manuscript), or elaborate on this item by providing additional information not in the ms, or briefly explain why the item is not applicable/relevant for your study

“Pharmacists across Germany were invited to participate by E-Mail […].”

1b-iv) RESULTS section in abstract must contain use data

Report number of participants enrolled/assessed in each group, the use/uptake of the intervention (e.g., attrition/adherence metrics, use over time, number of logins etc.), in addition to primary/secondary outcomes. (Note: Only report in the abstract what the main paper is reporting. If this information is missing from the main body of text, consider adding it)

subitem not at all important

1 ○

2 ○

3 ○

4 ●

5 ○

essential

Does your paper address subitem 1b-iv?

Copy and paste relevant sections from the manuscript abstract (include quotes in quotation marks "like this" to indicate direct quotes from your manuscript), or elaborate on this item by providing additional information not in the ms, or briefly explain why the item is not applicable/relevant for your study

„In total, 141 pharmacists were enrolled. In the Web-group (n=71 pharmacists), the quality score median was 32.8% (59.0 out of 180.0 points; IQR: 23.0–80.5). In the EVInews-group (n=70 pharmacists) the quality score median was significantly higher (85.3%; 153.5 points out of 180.0 points; *P*<.001) and the interquartile range (IQR) was smaller (IQR: 125.1–157.0)."

1b-v) CONCLUSIONS/DISCUSSION in abstract for negative trials

Conclusions/Discussions in abstract for negative trials: Discuss the primary outcome - if the trial is negative (primary outcome not changed), and the intervention was not used, discuss whether negative results are attributable to lack of uptake and discuss reasons. (Note: Only report in the abstract what the main paper is reporting. If this information is missing from the main body of text, consider adding it)

subitem not at all important

1 ○

2 ○

3 ○

4 ●

5 ○

essential

Does your paper address subitem 1b-v?

Copy and paste relevant sections from the manuscript abstract (include quotes in quotation marks "like this" to indicate direct quotes from your manuscript), or elaborate on this item by providing additional information not in the ms, or briefly explain why the item is not applicable/relevant for your study

This trial should not be considered as a negative trial, because both study groups did not yield negative results. The EVInews group however exceeded the median quality score of the control group. “The median quality score of the Web-group was poor and there was a significant difference in quality scores in favor of the EVInews-group. Pharmacists' web-based and self-medication related information sources often did not meet standard quality requirements and therefore showed a considerable variation in quality.”

INTRODUCTION

2a) In INTRODUCTION: Scientific background and explanation of rationale

2a-i) Problem and the type of system/solution

Describe the problem and the type of system/solution that is object of the study: intended as stand-alone intervention vs. incorporated in broader health care program? Intended for a particular patient population? Goals of the intervention, e.g., being more cost-effective to other interventions, replace or complement other solutions? (Note: Details about the intervention are provided in “Methods” under 5)

subitem not at all important

1 ○

2 ○

3 ○

4 ●

5 ○

essential

Does your paper address subitem 2a-i? *

Copy and paste relevant sections from the manuscript (include quotes in quotation marks "like this" to indicate direct quotes from your manuscript), or elaborate on this item by providing additional information not in the ms, or briefly explain why the item is not applicable/relevant for your study

“The treatment of clinical complaints with over-the-counter (OTC) products plays a substantial role in healthcare [1]. Community pharmacists are often the first and only point of contact within the healthcare system for self-medication related inquiries [2]. Self-medication counseling by pharmacists can contribute to a cost-effective, individualized, safe medical care on the patient level and consequently a relieve of the burden of the entire healthcare system [3, 4]. External evidence is required for well-grounded consultations and a key factor within the threefold evidence-based pharmacy (EbPharm) concept, which derived from evidence-based medicine (EbM) [5]. Apart from external evidence, the pharmacist’s practical experience and the patient’s preference should be considered when counseling [6, 7]. However, a lack of information sources, time and search skills have been identified as common barriers to EbPharm [8-18]. Consequently, various evidence-based information resources were introduced to reduce the evidence-to-practice gap. Among all information sources, electronic sources are of increasing importance for pharmacists [19, 20]. The EVInews project is an example for an electronic, evidence-based self-medication-related information platform for community pharmacists in Germany [21]. The majority of information sources are however not targeted for community pharmacists, especially not in the context of self-medication-counseling [22-30].
Little is known about the quality and the kind of electronic information sources that pharmacists use for evidence-based self-medication counseling.”

2a-ii) Scientific background, rationale: What is known about the (type of) system

Scientific background, rationale: What is known about the (type of) system that is the object of the study (be sure to discuss the use of similar systems for other conditions/diagnoses, if appropiate), motivation for the study, i.e. what are the reasons for and what is the context for this specific study, from which stakeholder viewpoint is the study performed, potential impact of findings [2]. Briefly justify the choice of the comparator.

subitem not at all important

1 ○

2 ○

3 ○

4 ●

5 ○

essential

Does your paper address subitem 2a-ii? *

Copy and paste relevant sections from the manuscript (include quotes in quotation marks "like this" to indicate direct quotes from your manuscript), or elaborate on this item by providing additional information not in the ms, or briefly explain why the item is not applicable/relevant for your study

"Little is known about the quality and the kind of electronic information sources that pharmacists use for evidence-based self-medication counseling. Information quality in the context of health is defined as a multidimensional concept that describes the fulfillment of individually predefined criteria [31, 32]. Several attempts have been made to measure the quality of electronic information sources through validated instruments [33-39]. Standard categories of these instruments address an information source's transparency, accuracy, completeness and readability for instance [40]. These instruments help identify valid information among the increasing body of available electronic data. Previous instruments, as a limitation, were only used for patient-oriented websites.
To date, literature about the quality of pharmacists' electronic information sources for self-medication counseling is scarce. Especially, studies that investigate the quality based on appropriate standard quality criteria of such electronic information sources are missing."

2b) In INTRODUCTION: Specific objectives or hypotheses

Does your paper address CONSORT subitem 2b? *

Copy and paste relevant sections from the manuscript (include quotes in quotation marks "like this" to indicate direct quotes from your manuscript), or elaborate on this item by providing additional information not in the ms, or briefly explain why the item is not applicable/relevant for your study

Study objectives
"Our aim was to investigate the quality of community pharmacists' web-based search results for self-medication related content when using the internet in comparison to the EVInews-database, based on an adjusted quality score for pharmacists. This study furthermore attempts to explore what kind of web-based sources are used for evidence-based self-medication counseling and how much time is needed under everyday practice conditions."

METHODS

3a) Description of trial design (such as parallel, factorial) including allocation ratio

Does your paper address CONSORT subitem 3a? *

Copy and paste relevant sections from the manuscript (include quotes in quotation marks "like this" to indicate direct quotes from your manuscript), or elaborate on this item by providing additional information not in the ms, or briefly explain why the item is not applicable/relevant for your study

"A quantitative, prospective, randomized, controlled online survey with an unblinded search-task and a parallel-study design was carried out between February 1st and April 30th 2021. The survey and data collection was conducted using SoSci Survey (Version 3.1.06). Participating pharmacists were automatically assigned through the randomization function of the software in a 1:1 allocation ratio to either study group after giving their informed consent."

3b) Important changes to methods after trial commencement (such as eligibility criteria), with reasons

Does your paper address CONSORT subitem 3b? *

Copy and paste relevant sections from the manuscript (include quotes in quotation marks "like this" to indicate direct quotes from your manuscript), or elaborate on this item by providing additional information not in the ms, or briefly explain why the item is not applicable/relevant for your study

Not applicable. No changes to methods were done after trial commencement.

3b-i) Bug fixes, Downtimes, Content Changes

Bug fixes, Downtimes, Content Changes: ehealth systems are often dynamic systems. A description of changes to methods therefore also includes important changes made on the intervention or comparator during the trial (e.g., major bug fixes or changes in the functionality or content) (5-iii) and other “unexpected events” that may have influenced study design such as staff changes, system failures/downtimes, etc. [2].

subitem not at all important

1 ○

2 ○

3 ○

4 ●

5 ○

essential

Does your paper address subitem 3b-i?

Copy and paste relevant sections from the manuscript (include quotes in quotation marks "like this" to indicate direct quotes from your manuscript), or elaborate on this item by providing additional information not in the ms, or briefly explain why the item is not applicable/relevant for your study

Not applicable. No technical changes were undertaken after trial commencement. The EVInews-database’s content was updated during the conduction of the trial. This content was however irrelevant for the participants.

4a) Eligibility criteria for participants

Does your paper address CONSORT subitem 4a? *

Copy and paste relevant sections from the manuscript (include quotes in quotation marks "like this" to indicate direct quotes from your manuscript), or elaborate on this item by providing additional information not in the ms, or briefly explain why the item is not applicable/relevant for your study

“Inclusion criteria: Participants had to be licensed “pharmacists”.”

4a-i) Computer / Internet literacy

Computer / Internet literacy is often an implicit “de facto” eligibility criterion - this should be explicitly clarified.

subitem not at all important

1 ○

2 ○

3 ●

4 ○

5 ○

essential

Does your paper address subitem 4a-i?

Copy and paste relevant sections from the manuscript (include quotes in quotation marks "like this" to indicate direct quotes from your manuscript), or elaborate on this item by providing additional information not in the ms, or briefly explain why the item is not applicable/relevant for your study

Not relevant. “Participants of both study groups were asked to verify 6 health-related statements from 2 typical self-medication indications (s. multimedia appendix Supplement 1) with a quote and the corresponding information source (search-task). Out of the 6 statements, 3 belonged to the self-medication indications recurrent Herpes labials and female Androgenetic Alopecia respectively. The indications were chosen randomly from topics available in the EVInews-database.
A video and a short written summary was presented to all participants to inform about the study protocol. The 3 statements of each indication were then displayed in an alternating order based on the software’s Rotate Blocks function to the participants. This was used to prevent learning effects and therefore a distortion of the results. Participants were then instructed to copy and paste their quote and link of the information source that proved or disproved the correctness of each statement in designated text boxes.” The users were asked to complete a search-task under routine conditions, which depicts a typical counseling scenario in community pharmacies. Since a basic computer/internet literacy is fundamental for community pharmacists to run daily operations, it can be assumed that participants already possessed these skills for the completion of the search task.

4a-ii) Open vs. closed, web-based vs. face-to-face assessments:

Open vs. closed, web-based vs. face-to-face assessments: Mention how participants were recruited (online vs. offline), e.g., from an open access website or from a clinic, and clarify if this was a purely web-based trial, or there were face-to-face components (as part of the intervention or for assessment), i.e., to what degree got the study team to know the participant. In online-only trials, clarify if participants were quasi-anonymous and whether having multiple identities was possible or whether technical or logistical measures (e.g., cookies, email confirmation, phone calls) were used to detect/prevent these.

subitem not at all important

1 ○

2 ○

3 ○

4 ●

5 ○

essential

Does your paper address subitem 4a-ii? *

Copy and paste relevant sections from the manuscript (include quotes in quotation marks "like this" to indicate direct quotes from your manuscript), or elaborate on this item by providing additional information not in the ms, or briefly explain why the item is not applicable/relevant for your study

“Community pharmacists across Germany within 5 different Federal Pharmacy Chambers (Baden-Wuerttemberg, Bavaria, Mecklenburg-Western Pomerania, Lower Saxony, Saxony) were invited via E-Mail to participate online and anonymously without any incentives. For this purpose, invitations containing the survey were sent by E-mail to all community pharmacies that agreed to receive survey invitations and were listed in email-mailing list of each Federal Pharmacy Chamber. The completeness rate of the survey and time needed for completion was assessed through the SoSci survey software. A reminder E-Mail was sent after 1 month in case less than 15 participants within a pharmacy chamber enrolled.”

4a-iii) Information giving during recruitment

Information given during recruitment. Specify how participants were briefed for recruitment and in the informed consent procedures (e.g., publish the informed consent documentation as appendix, see also item X26), as this information may have an effect on user self-selection, user expectation and may also bias results.

subitem not at all important

1 ○

2 ○

3 ●

4 ○

5 ○

essential

Does your paper address subitem 4a-iii?

Copy and paste relevant sections from the manuscript (include quotes in quotation marks "like this" to indicate direct quotes from your manuscript), or elaborate on this item by providing additional information not in the ms, or briefly explain why the item is not applicable/relevant for your study

“Community pharmacists across Germany within 5 different Federal Pharmacy Chambers (Baden-Wuerttemberg, Bavaria, Mecklenburg-Western Pomerania, Lower Saxony, Saxony) were invited via E-Mail to participate online and anonymously without any incentives. For this purpose, invitations containing the survey were sent by E-mail to all community pharmacies that agreed to receive survey invitations and were listed in email-mailing list of each Federal Pharmacy Chamber. The completeness rate of the survey and time needed for completion was assessed through the SoSci survey software.”

Participants had to give their informed consent. Data processing and handling met the European data protection law standards. The consent statements were not included within the Appendix but are as follows: “Clarification about participation in the survey
The data collection is anonymous and does not allow any conclusion about your identity. All data will be treated confidentially and will only be viewed by staff involved in the project and will only be used for scientific purposes. I agree to the processing of my anonymous data within the framework of the project.”

4b) Settings and locations where the data were collected

Does your paper address CONSORT subitem 4b? *

Copy and paste relevant sections from the manuscript (include quotes in quotation marks "like this" to indicate direct quotes from your manuscript), or elaborate on this item by providing additional information not in the ms, or briefly explain why the item is not applicable/relevant for your study

“Community pharmacists across Germany within 5 different Federal Pharmacy Chambers (Baden-Wuerttemberg, Bavaria, Mecklenburg-Western Pomerania, Lower Saxony, Saxony) were invited via E-Mail to participate online and anonymously without any incentives.”

4b-i) Report if outcomes were (self-)assessed through online questionnaires

Clearly report if outcomes were (self-)assessed through online questionnaires (as common in web-based trials) or otherwise.

subitem not at all important

1 ○

2 ○

3 ○

4 ○

5 ●

essential

Does your paper address subitem 4b-i? *

Copy and paste relevant sections from the manuscript (include quotes in quotation marks "like this" to indicate direct quotes from your manuscript), or elaborate on this item by providing additional information not in the ms, or briefly explain why the item is not applicable/relevant for your study

“Outcome parameters
The primary outcome parameter was the difference in quality scores between the study groups. Secondary outcome parameters included a subgroup analysis, quality scores of the rating scheme categories transparency, quality of evidence and usability, the characterization of web-based information sources, a correlational analysis of sociodemographic characteristics and quality scores, a self-evaluation of search skills by the participants and assessment of the time needed to perform the search-task.”

4b-ii) Report how institutional affliations are displayed

Report how institutional afiliations are displayed to potential participants [on ehealth media], as afiliations with prestigious hospitals or universities may affect volunteer rates, use, and reactions with regards to an intervention. (Not a required item – describe only if this may bias results)

subitem not at all important

- 1 ○
- 2 ●
- 3 ○
- 4 ○
- 5 ○

essential

Does your paper address subitem 4b-ii?

Copy and paste relevant sections from the manuscript (include quotes in quotation marks "like this" to indicate direct quotes from your manuscript), or elaborate on this item by providing additional information not in the ms, or briefly explain why the item is not applicable/relevant for your study

The logo of the Medical Faculty of the University of Leipzig as well as contact details of the staff involved in the study conduction were displayed on the survey pages. The affiliations were not expected to impact the study participant’s behaviour, other than implying trustworthiness and safety of the survey’s content and link. The participant’s data was furthermore collected anonymously. Since we did not regard the disclosure as a potential threat to bias, we did not include this item in the manuscript.

5) The interventions for each group with suffcient details to allow replication, including how and when they were actually administered

5-i) Mention names, credential, affiliations of the developers, sponsors, and owners

Mention names, credential, afiliations of the developers, sponsors, and owners [6] (if authors/evaluators are owners or developer of the software, this needs to be declared in a “Conflict of interest” section or mentioned elsewhere in the manuscript).

subitem not at all important

1 ○

2 ○

3 ●

4 ○

5 ○

essential

Does your paper address subitem 5-i?

Copy and paste relevant sections from the manuscript (include quotes in quotation marks "like this" to indicate direct quotes from your manuscript), or elaborate on this item by providing additional information not in the ms, or briefly explain why the item is not applicable/relevant for your study

We did not create or maintain the web-based information sources of choice that participants within the web-group used. Since Jennifer Alexa is one of the authors of the EVInews database’s content, her authorship is mentioned as a conflict of interest.

“Third party funding
Jennifer Alexa received third party funding from the ABDA—Federal Union of German Associations of Pharmacists (Berlin, Germany). Matthias Richter received third party funding from the ABDA—Federal Union of German Associations of Pharmacists (Berlin, Germany).”

“Conflicts of interest:
Conflicts of interest:
Jennifer Maria Alexa is one of the EVInews newsletter authors and composed newsletters under the guidance of Thilo Bertsche. All authors developed the rating scheme.”

Information regarding the EVInews information platform is revealed within the methods section. “Independent pharmacists from the Department of Clinical Pharmacy at Leipzig University compose the Newsletters with editorial assistance from AVOXA—Media Group German Pharmacist GmbH.”

5-ii) Describe the history/development process

Describe the history/development process of the application and previous formative evaluations (e.g., focus groups, usability testing), as these will have an impact on adoption/use rates and help with interpreting results.

subitem not at all important

1 ○

2 ○

3 ○

4 ●

5 ○

essential

Does your paper address subitem 5-ii?

Copy and paste relevant sections from the manuscript (include quotes in quotation marks "like this" to indicate direct quotes from your manuscript), or elaborate on this item by providing additional information not in the ms, or briefly explain why the item is not applicable/relevant for your study

“Survey and quality score development
In order to ensure feasibility and exclude comprehension difficulties, pretests and pilot tests were conducted between October 26 2020 to February 14 2021. The initial pretests were carried out with 23 Pharmacists. The first 12 pretests were executed using the think-aloud method, the remaining using the standard observation pretest. After a revision and adjustment of the survey and rating scheme, a pilot test with 37 pharmacists and advanced pharmacy students as pre-testers was performed resulting in minor modifications. Furthermore, to reduce subjective judgement on each quote’s suitability (item 4 d within the rating scheme), 1359 relevant keywords in English and German were identified during the pretest phase and used for a summative content analysis. The frequency of these predefined key words was measured through the Excel count function for each quote. To assess whether difficulties occurred during the completion of the search-task, participants were asked to rate the clarity of this survey and give qualitative feedback if desired. Data generated by the pre- and pilot-test was not included in this study.”

5-iii) Revisions and updating

Revisions and updating. Clearly mention the date and/or version number of the application/intervention (and comparator, if applicable) evaluated, or describe whether the intervention underwent major changes during the evaluation process, or whether the development and/or content was “frozen” during the trial. Describe dynamic components such as news feeds or changing content which may have an impact on the replicability of the intervention (for unexpected events see item 3b).

subitem not at all important

1 ○

2 ○

3 ●

4 ○

5 ○

essential

Does your paper address subitem 5-iii?

Copy and paste relevant sections from the manuscript (include quotes in quotation marks "like this" to indicate direct quotes from your manuscript), or elaborate on this item by providing additional information not in the ms, or briefly explain why the item is not applicable/relevant for your study

The existing newsletters and database entries remained unchanged. However, new newsletters and database entries were added on a regular basis. This did not affect the conduction of this study as the topics were pre-determined and were not updated during the conduction of the study. All utilized software and software versions for the data analysis are named within the Methods section.

“The survey and data collection was conducted using SoSci Survey (Version 3.1.06).”

“The analysis was performed using Microsoft Office Excel (version 2105 and 2016, Microsoft Corporation, Redmond, Washington, U.S.A.) and IBM® SPSS® Statistics version 25 and 26 (IBM Corporation, Armonk, New York, U.S.A.).”

5-iv) Quality assurance methods

Provide information on quality assurance methods to ensure accuracy and quality of information provided [1], if applicable.

subitem not at all important

1 ○

2 ○

3 ○

4 ●

5 ○

essential

Does your paper address subitem 5-iv?

Copy and paste relevant sections from the manuscript (include quotes in quotation marks "like this" to indicate direct quotes from your manuscript), or elaborate on this item by providing additional information not in the ms, or briefly explain why the item is not applicable/relevant for your study

“Quality assurance means
Survey and quality score development
In order to ensure feasibility and exclude comprehension difficulties, pretests and pilot tests were conducted between October 26 2020 to February 14 2021. The initial pretests were carried out with 23 Pharmacists. The first 12 pretests were executed using the think-aloud method, the remaining using the standard observation pretest. After a revision and adjustment of the survey and rating scheme, a pilot test with 37 pharmacists and advanced pharmacy students as pre-testers was performed resulting in minor modifications. Furthermore, to reduce subjective judgement on each quote’s suitability (item 4 d within the rating scheme), 1359 relevant keywords in English and German were identified during the pretest phase and used for a summative content analysis. The frequency of these predefined key words was measured through the Excel count function for each quote. To assess whether difficulties occurred during the completion of the search-task, participants were asked to rate the clarity of this survey and give qualitative feedback if desired. Data generated by the pre- and pilot-test was not included in this study.”

5-v) Ensure replicability by publishing the source code, and/or providing screenshots/screen-capture video, and/or providing flowcharts of the algorithms used

Ensure replicability by publishing the source code, and/or providing screenshots/screen-capture video, and/or providing flowcharts of the algorithms used. Replicability (i.e., other researchers should in principle be able to replicate the study) is a hallmark of scientific reporting.

subitem not at all important

1 ○
2 ○
3 ●
4 ○
5 ○

essential

Does your paper address subitem 5-v?

Copy and paste relevant sections from the manuscript (include quotes in quotation marks "like this" to indicate direct quotes from your manuscript), or elaborate on this item by providing additional information not in the ms, or briefly explain why the item is not applicable/relevant for your study

The rating scheme is enclosed in the multimedia appendix.
“An adjusted quality score was used to assess the information sources that were used for the search-task. Therefore, a rating scheme for the evaluation of the search-task was created (s. multimedia appendix Supplement 2).”

5-vi) Digital preservation

Digital preservation: Provide the URL of the application, but as the intervention is likely to change or disappear over the course of the years; also make sure the intervention is archived (Internet Archive, webcitation.org, and/or publishing the source code or screenshots/videos alongside the article). As pages behind login screens cannot be archived, consider creating demo pages which are accessible without login.

subitem not at all important

1 ○

2 ○

3 ●

4 ○

5 ○

essential

Does your paper address subitem 5-vi?

Copy and paste relevant sections from the manuscript (include quotes in quotation marks "like this" to indicate direct quotes from your manuscript), or elaborate on this item by providing additional information not in the ms, or briefly explain why the item is not applicable/relevant for your study

Participants within the EVInews-group were advised to use the EVInews database only. The database was accessible through the link: https://www.evinews.de/index.php?id=login.

“The EVInews-group accessed the database via the link: https://www.evinews.de/index.php?id=login and a test-account.”

5-vii) Access

Access: Describe how participants accessed the application, in what setting/context, if they had to pay (or were paid) or not, whether they had to be a member of specific group. If known, describe how participants obtained “access to the platform and Internet” [1]. To ensure access for editors/reviewers/readers, consider to provide a “backdoor” login account or demo mode for reviewers/readers to explore the application (also important for archiving purposes, see vi).

subitem not at all important

1 ○

2 ○

3 ○

4 ●

5 ○

essential

Does your paper address subitem 5-vii? *

Copy and paste relevant sections from the manuscript (include quotes in quotation marks "like this" to indicate direct quotes from your manuscript), or elaborate on this item by providing additional information not in the ms, or briefly explain why the item is not applicable/relevant for your study

The web-based information sources of choice were accessible for the participants within the web-group, since they were asked to use the web-based sources they would normally use.

“The EVInews-group accessed the database via the link: https://www.evinews.de/index.php?id=login and a test-account.”

The EVInews database is free of charge as mentioned within the methods section.
“The EVInews-database
The EVInews project was launched in 2017 and is based on the resolution of the Federal Union of German Associations of Pharmacists (ABDA) to help implement EbPharm into everyday practice. It consists of a website with general EbPharm information, monthly published Newsletters and a corresponding document-oriented database, which are free of charge. Independent pharmacists from the Department of Clinical Pharmacy at Leipzig University compose the Newsletters with editorial assistance from AVOXA—Media Group German Pharmacist GmbH.”

5-viii) Mode of delivery, features/functionalities/components of the intervention and comparator, and the theoretical framework

Describe mode of delivery, features/functionalities/components of the intervention and comparator, and the theoretical framework [6] used to design them (instructional strategy [1], behaviour change techniques, persuasive features, etc., see e.g., [7, 8] for terminology). This includes an in-depth description of the content (including where it is coming from and who developed it) [1]," whether [and how] it is tailored to individual circumstances and allows users to track their progress and receive feedback" [6]. This also includes a description of communication delivery channels and – if computer-mediated communication is a component – whether communication was synchronous or asynchronous [6]. It also includes information on presentation strategies [1], including page design principles, average amount of text on pages, presence of hyperlinks to other resources, etc. [1].

subitem not at all important

1 ○

2 ○

3 ○

4 ●

5 ○

essential

Does your paper address subitem 5-viii? *

Copy and paste relevant sections from the manuscript (include quotes in quotation marks "like this" to indicate direct quotes from your manuscript), or elaborate on this item by providing additional information not in the ms, or briefly explain why the item is not applicable/relevant for your study

“Rating scheme
Rating scheme criteria were chosen and adapted based on validated quality scores and questionnaires reported in the literature [35-37] as well as previous findings [32, 39, 41-43]. The rating scheme consists of 18 nominal to ordinal scaled criteria that address predefined quality standards. We selected existing and added new criteria that were relevant for the search-task. The rating scheme criteria were grouped together in the categories “transparency”, “quality of evidence” and “usability” for the information sources and “accuracy” for the evaluation of the quotes. Out of all items, 12 possessed a dichotomous response format and 6 an ordinal response format.”

“Survey and quality score development
In order to ensure feasibility and exclude comprehension difficulties, pretests and pilot tests were conducted between October 26 2020 to February 14 2021. The initial pretests were carried out with 23 Pharmacists. The first 12 pretests were executed using the think-aloud method, the remaining using the standard observation pretest. After a revision and adjustment of the survey and rating scheme, a pilot test with 37 pharmacists and advanced pharmacy students as pre-testers was performed resulting in minor modifications. Furthermore, to reduce subjective judgement on each quote’s suitability (item 4 d within the rating scheme), 1359 relevant keywords in English and German were identified during the pretest phase and used for a summative content analysis. The frequency of these predefined key words was measured through the Excel count function for each quote. To assess whether difficulties occurred during the completion of the search-task, participants were asked to rate the clarity of this survey and give qualitative feedback if desired.

5-ix) Describe use parameters

Describe use parameters (e.g., intended “doses” and optimal timing for use). Clarify what instructions or recommendations were given to the user, e.g., regarding timing, frequency, heaviness of use, if any, or was the intervention used ad libitum.

subitem not at all important

- 1 ○
- 2 ●
- 3 ○
- 4 ○
- 5 ○

essential

Does your paper address subitem 5-ix?

Copy and paste relevant sections from the manuscript (include quotes in quotation marks "like this" to indicate direct quotes from your manuscript), or elaborate on this item by providing additional information not in the ms, or briefly explain why the item is not applicable/relevant for your study

Not applicable. The survey completion and therefore usage of the allocated information sources was done at any time between “February 1st and April 30th 2021.“ The intervention and control was therefore applied ad libitum.

5-x) Clarify the level of human involvement

Clarify the level of human involvement (care providers or health professionals, also technical assistance) in the e-intervention or as co-intervention (detail number and expertise of professionals involved, if any, as well as “type of assistance offered, the timing and frequency of the support, how it is initiated, and the medium by which the assistance is delivered”. It may be necessary to distinguish between the level of human involvement required for the trial, and the level of human involvement required for a routine application outside of a RCT setting (discuss under item 21 – generalizability).

subitem not at all important

1 ○

2 ○

3 ●

4 ○

5 ○

essential

Does your paper address subitem 5-x?

Copy and paste relevant sections from the manuscript (include quotes in quotation marks "like this" to indicate direct quotes from your manuscript), or elaborate on this item by providing additional information not in the ms, or briefly explain why the item is not applicable/relevant for your study

The data collected was anonymous. There was no human interaction between the participants and staff involved in the study conduction and analysis.

5-xi) Report any prompts/reminders used

Report any prompts/reminders used: Clarify if there were prompts (letters, emails, phone calls, SMS) to use the application, what triggered them, frequency etc. It may be necessary to distinguish between the level of prompts/reminders required for the trial, and the level of prompts/reminders for a routine application outside of a RCT setting (discuss under item 21 – generalizability).

subitem not at all important

- 1 ○
- 2 ●
- 3 ○
- 4 ○
- 5 ○

essential

Does your paper address subitem 5-xi? *

Copy and paste relevant sections from the manuscript (include quotes in quotation marks "like this" to indicate direct quotes from your manuscript), or elaborate on this item by providing additional information not in the ms, or briefly explain why the item is not applicable/relevant for your study

“A reminder E-Mail was sent after 1 month in case less than 15 participants within a pharmacy chamber enrolled.” The collected data was anonymous, therefore no individual participant or potential, individual participant did receive a reminder E-Mail.

5-xii) Describe any co-interventions (incl. training/support)

Describe any co-interventions (incl. training/support): Clearly state any interventions that are provided in addition to the targeted eHealth intervention, as ehealth intervention may not be designed as stand-alone intervention. This includes training sessions and support [1]. It may be necessary to distinguish between the level of training required for the trial, and the level of training for a routine application outside of a RCT setting (discuss under item 21 – generalizability.

subitem not at all important

1 ○

2 ○

3 ○

4 ●

5 ○

essential

Does your paper address subitem 5-xii? *

Copy and paste relevant sections from the manuscript (include quotes in quotation marks "like this" to indicate direct quotes from your manuscript), or elaborate on this item by providing additional information not in the ms, or briefly explain why the item is not applicable/relevant for your study

Both information tools consisting of the web-based information sources of choice and the EVInews database. Therefore, no co-interventions are mentioned. There were no non-web-based components in this study.
“A video and a short written summary was presented to all participants to inform about the study protocol. The 3 statements of each indication were then displayed in an alternating order based on the software’s Rotate Blocks function to the participants. This was used to prevent learning effects and therefore a distortion of the results. Participants were then instructed to copy and paste their quote and link of the information source that proved or disproved the correctness of each statement in designated text boxes.”

6a) Completely defined pre-specified primary and secondary outcome measures, including how and when they were assessed

Does your paper address CONSORT subitem 6a? *

Copy and paste relevant sections from the manuscript (include quotes in quotation marks "like this" to indicate direct quotes from your manuscript), or elaborate on this item by providing additional information not in the ms, or briefly explain why the item is not applicable/relevant for your study

All analysis was done after the termination of the study.
“The primary outcome parameter was the difference in quality scores between the study groups. Secondary outcome parameters included a subgroup analysis, quality scores of the rating scheme categories transparency, quality of evidence and usability, the characterization of web-based information sources, a correlational analysis of sociodemographic characteristics and quality scores, a self-evaluation of search skills by the participants and assessment of the time needed to perform the search-task.”

6a-i) Online questionnaires: describe if they were validated for online use and apply
CHERRIES items to describe how the questionnaires were designed/deployed

If outcomes were obtained through online questionnaires, describe if they were validated for online use and apply CHERRIES items to describe how the questionnaires were designed/deployed [9].

subitem not at all important

1 ○

2 ○

3 ●

4 ○

5 ○

essential

Does your paper address subitem 6a-i?

Copy and paste relevant sections from manuscript text

A questionnaire validation was not done prior to the study conduction, which is also mentioned as a limitation of this study. However extensive pretests and pilot tests were performed

“In order to ensure feasibility and exclude comprehension difficulties, pretests and pilot tests were conducted between October 26 2020 to February 14 2021. The initial pretests were carried out with 23 Pharmacists. The first 12 pretests were executed using the think-aloud method, the remaining using the standard observation pretest. After a revision and adjustment of the survey and rating scheme, a pilot test with 37 pharmacists and advanced pharmacy students as pre-testers was performed resulting in minor modifications.”

6a-ii) Describe whether and how “use” (including intensity of use/dosage) was defined/measured/monitored

Describe whether and how “use” (including intensity of use/dosage) was defined/measured/monitored (logins, logfile analysis, etc.). Use/adoption metrics are important process outcomes that should be reported in any ehealth trial.

subitem not at all important

1 ○

2 ○

3 ○

4 ●

5 ○

essential

Does your paper address subitem 6a-ii?

Copy and paste relevant sections from manuscript text

Not applicable. The frequency of use was not measured since participants were able to use the assigned information sources ad libitum throughout the trial duration and data obtained by this study was anonymous.

“A quantitative, prospective, randomized, controlled online survey with an unblinded search-task and a parallel-study design was carried out between February 1st and April 30th 2021. The survey and data collection was conducted using SoSci Survey (Version 3.1.06). Participating pharmacists were automatically assigned through the randomization function of the software in a 1:1 allocation ratio to either study group after giving their informed consent.”

6a-iii) Describe whether, how, and when qualitative feedback from participants was obtained

Describe whether, how, and when qualitative feedback from participants was obtained (e.g., through emails, feedback forms, interviews, focus groups).

subitem not at all important

1 ○

2 ○

3 ●

4 ○

5 ○

essential

Does your paper address subitem 6a-iii?

Copy and paste relevant sections from manuscript text

“To assess whether difficulties occurred during the completion of the search-task, participants were asked to rate the clarity of this survey and give qualitative feedback if desired.”

6b) Any changes to trial outcomes after the trial commenced, with reasons

Does your paper address CONSORT subitem 6b? *

Copy and paste relevant sections from the manuscript (include quotes in quotation marks "like this" to indicate direct quotes from your manuscript), or elaborate on this item by providing additional information not in the ms, or briefly explain why the item is not applicable/relevant for your study

Not applicable. No changes to trial outcomes were done.

7a) How sample size was determined

NPT: When applicable, details of whether and how the clustering by care provides or centers was addressed

7a-i) Describe whether and how expected attrition was taken into account when calculating the sample size

Describe whether and how expected attrition was taken into account when calculating the sample size.

subitem not at all important

1 ○

2 ○

3 ○

4 ●

5 ○

essential

Does your paper address subitem 7a-i?

Copy and paste relevant sections from manuscript title (include quotes in quotation marks "like this" to indicate direct quotes from your manuscript), or elaborate on this item by providing additional information not in the ms, or briefly explain why the item is not applicable/relevant for your study

“Sample size
The estimated sample size was calculated using the G*Power Software (version 3.1). No information regarding the primary target distribution was available. Based on the results of a pilot test, a double-sided Mann-Whitney-U-test for non-parametric, independent samples was used a priori to determine the required sample size for a significant difference in quality scores. At a significance level of α=.05 and a confidence interval of 95%, a sample size of at least 44 pharmacists per group would provide a power of 1-ß=.80. We estimated attrition to be 50% and consequently strived for a greater sample size.”

7b) When applicable, explanation of any interim analyses and stopping guidelines

Does your paper address CONSORT subitem 7b? *

Copy and paste relevant sections from the manuscript (include quotes in quotation marks "like this" to indicate direct quotes from your manuscript), or elaborate on this item by providing additional information not in the ms, or briefly explain why the item is not applicable/relevant for your study

Not applicable. No interim analysis was done. Stopping guidelines were not necessary as participants used the assigned information sources ad libitum.
"**Data analysis**
Only data of participants, who processed the search-task and did not violate the study protocol, was included in the data analysis. The analysis was performed using Microsoft Office Excel […]."

8a) Method used to generate the random allocation sequence

NPT: When applicable, how care providers were allocated to each trial group

Does your paper address CONSORT subitem 8a? *

Copy and paste relevant sections from the manuscript (include quotes in quotation marks "like this" to indicate direct quotes from your manuscript), or elaborate on this item by providing additional information not in the ms, or briefly explain why the item is not applicable/relevant for your study

"The survey and data collection was conducted using SoSci Survey (Version 3.1.06). Participating pharmacists were automatically assigned through the randomization function of the software in a 1:1 allocation ratio to either study group after giving their informed consent."

8b) Type of randomisation; details of any restriction (such as blocking and block size)

Does your paper address CONSORT subitem 8b? *

Copy and paste relevant sections from the manuscript (include quotes in quotation marks "like this" to indicate direct quotes from your manuscript), or elaborate on this item by providing additional information not in the ms, or briefly explain why the item is not applicable/relevant for your study

"The survey and data collection was conducted using SoSci Survey (Version 3.1.06). Participating pharmacists were automatically assigned through the randomization function of the software in a 1:1 allocation ratio to either study group after giving their informed consent."

9) Mechanism used to implement the random allocation sequence (such as sequentially numbered containers), describing any steps taken to conceal the sequence until interventions were assigned

Does your paper address CONSORT subitem 9? *

Copy and paste relevant sections from the manuscript (include quotes in quotation marks "like this" to indicate direct quotes from your manuscript), or elaborate on this item by providing additional information not in the ms, or briefly explain why the item is not applicable/relevant for your study

Random allocation sequence was generated automatically by the SoSci Survey Software as mentioned previously.
“The survey and data collection was conducted using SoSci Survey (Version 3.1.06). Participating pharmacists were automatically assigned through the randomization function of the software in a 1:1 allocation ratio to either study group after giving their informed consent.”

10) Who generated the random allocation sequence, who enrolled participants, and who assigned participants to interventions

Does your paper address CONSORT subitem 10? *

Copy and paste relevant sections from the manuscript (include quotes in quotation marks "like this" to indicate direct quotes from your manuscript), or elaborate on this item by providing additional information not in the ms, or briefly explain why the item is not applicable/relevant for your study

Random allocation sequence was generated automatically by the SoSci Survey Software as mentioned previously.
“The survey and data collection was conducted using SoSci Survey (Version 3.1.06). Participating pharmacists were automatically assigned through the randomization function of the software in a 1:1 allocation ratio to either study group after giving their informed consent.”

11a) If done, who was blinded after assignment to interventions (for example, participants, care providers, those assessing outcomes) and how

NPT: Whether or not administering co-interventions were blinded to group assignment

11a-i) Specify who was blinded, and who wasn’t

Specify who was blinded, and who wasn’t. Usually, in web-based trials it is not possible to blind the participants [1, 3] (this should be clearly acknowledged), but it may be possible to blind outcome assessors, those doing data analysis or those administering co-interventions (if any).

subitem not at all important

1 ○

2 ○

3 ○

4 ●

5 ○

essential

Does your paper address subitem 11a-i? *

Copy and paste relevant sections from the manuscript (include quotes in quotation marks "like this" to indicate direct quotes from your manuscript), or elaborate on this item by providing additional information not in the ms, or briefly explain why the item is not applicable/relevant for your study

Not applicable. Blinding was not possible since the information sources that participants used automatically revealed to which study group they belonged.

The lack of blinding is therefore mentioned among the study limitations.

“Raters and participants were not blinded. Since the type of information sources the users were allowed to use was predefined, blinding was not possible. In order to minimize subjective evaluation, a quantitative approach for the quality evaluation with objective criteria was chosen to ascertain a robust rating performance.”

11a-ii) Discuss e.g., whether participants knew which intervention was the “intervention of interest” and which one was the “comparator”

Informed consent procedures (4a-ii) can create biases and certain expectations - discuss e.g., whether participants knew which intervention was the “intervention of interest” and which one was the “comparator”.

subitem not at all important

1 ○

2 ○

3 ●

4 ○

5 ○

essential

Does your paper address subitem 11a-ii?

Copy and paste relevant sections from the manuscript (include quotes in quotation marks "like this" to indicate direct quotes from your manuscript), or elaborate on this item by providing additional information not in the ms, or briefly explain why the item is not applicable/relevant for your study

Not applicable. Two different kinds of information sources were tested throughout this study. All instructions were formulated in a neutral way. This study furthermore also possesses an exploratory trait, since the type of web-based information sources that community pharmacists use for the daily self-medication-counseling practice was investigated.

11b) If relevant, description of the similarity of interventions

(this item is usually not relevant for ehealth trials as it refers to similarity of a placebo or sham intervention to a active medication/intervention)

Does your paper address CONSORT subitem 11b? *

Copy and paste relevant sections from the manuscript (include quotes in quotation marks "like this" to indicate direct quotes from your manuscript), or elaborate on this item by providing additional information not in the ms, or briefly explain why the item is not applicable/relevant for your study

Not applicable.

12a) Statistical methods used to compare groups for primary and secondary outcomes

NPT: When applicable, details of whether and how the clustering by care providers or centers was addressed

Does your paper address CONSORT subitem 12a? *

Copy and paste relevant sections from the manuscript (include quotes in quotation marks "like this" to indicate direct quotes from your manuscript), or elaborate on this item by providing additional information not in the ms, or briefly explain why the item is not applicable/relevant for your study

“Data analysis
[…] Results for the primary target parameter (quality score) were expressed as median and first and third quartile (Q25/Q75). They were compared using a two-sided Mann-Whitney-U-test for independent samples. The comparison of quality sub-scores was performed likewise with a Bonferroni correction. Descriptive statistics were used to summarize the responses. Spearman’s rank correlation coefficients were calculated to investigate the relationship between quality scores and sociodemographic data.”

12a-i) Imputation techniques to deal with attrition / missing values

Imputation techniques to deal with attrition / missing values: Not all participants will use the intervention/comparator as intended and attrition is typically high in ehealth trials.
Specify how participants who did not use the application or dropped out from the trial were treated in the statistical analysis (a complete case analysis is strongly discouraged, and simple imputation techniques such as LOCF may also be problematic [4]).

subitem not at all important

1 ○

2 ○

3 ○

4 ●

5 ○

essential

Does your paper address subitem 12a-i? *

Copy and paste relevant sections from the manuscript (include quotes in quotation marks "like this" to indicate direct quotes from your manuscript), or elaborate on this item by providing additional information not in the ms, or briefly explain why the item is not applicable/relevant for your study

Not applicable. No imputation techniques were used.

12b) Methods for additional analyses, such as subgroup analyses and adjusted analyses

Does your paper address CONSORT subitem 12b? *

Copy and paste relevant sections from the manuscript (include quotes in quotation marks "like this" to indicate direct quotes from your manuscript), or elaborate on this item by providing additional information not in the ms, or briefly explain why the item is not applicable/relevant for your study

"**Study subgroups**
Since not all participants completed the search task of the survey, a subgroup analysis of these participants was performed as demonstrated in Figure 1. Participants who chose at least 1 answer option including "I do not know" within the search-task were defined as 'met the eligibility criteria ("all")'. Participants, who chose 1 answer option and verified at least 1 statement with a quote and source were defined as 'completed search-task partially (subgroup "completed partially").' Participants who verified all 6 health-related statements were defined as 'completed search-task fully (subgroup "completed fully")' […]."

"Outcome parameters
Secondary outcome parameters included a subgroup analysis, quality scores of the rating scheme categories […]."

X26) REB/IRB Approval and Ethical Considerations [recommended as subheading under "Methods"] (not a CONSORT item)

X26-i) Comment on ethics committee approval

subitem not at all important

1 ○

2 ○

3 ●

4 ○

5 ○

essential

Does your paper address subitem X26-i?

Copy and paste relevant sections from the manuscript (include quotes in quotation marks "like this" to indicate direct quotes from your manuscript), or elaborate on this item by providing additional information not in the ms, or briefly explain why the item is not applicable/relevant for your study

"**Ethics approval**
Ethical approval for this study was granted by the ethics committee of the Medical Faculty of the Leipzig University (541/20-ek) on the 5th of December 2020."

x26-ii) Outline informed consent procedures

Outline informed consent procedures e.g., if consent was obtained offline or online (how? Checkbox, etc.?), and what information was provided (see 4a-ii). See [6] for some items to be included in informed consent documents.

subitem not at all important

1 ○

2 ●

3 ○

4 ○

5 ○

essential

Does your paper address subitem X26-ii?

Copy and paste relevant sections from the manuscript (include quotes in quotation marks "like this" to indicate direct quotes from your manuscript), or elaborate on this item by providing additional information not in the ms, or briefly explain why the item is not applicable/relevant for your study

“Participating pharmacists were automatically assigned through the randomization function of the software in a 1:1 allocation ratio to either study group after giving their informed consent.”

Participants were invited to participate voluntarily. The original text regarding informed consent within the questionnaire was as follows:

The data collection is anonymous and does not enable any conclusion about your identity. All data will be treated confidentially and will only be viewed by staff involved in the project and used exclusively for scientific purposes.
I agree to the processing of my anonymous data within the framework of this project.

As this is standardized information, we did not expect to obtain any additional information by publishing it in the appendices. This study was conducted in Germany and therefore needs to fulfill German data protection laws.

X26-iii) Safety and security procedures

Safety and security procedures, incl. privacy considerations, and any steps taken to reduce the likelihood or detection of harm (e.g., education and training, availability of a hotline)

subitem not at all important

- 1 ○
- 2 ○
- 3 ●
- 4 ○
- 5 ○

essential

Does your paper address subitem X26-iii?

Copy and paste relevant sections from the manuscript (include quotes in quotation marks "like this" to indicate direct quotes from your manuscript), or elaborate on this item by providing additional information not in the ms, or briefly explain why the item is not applicable/relevant for your study

Not applicable. Privacy was granted since the data obtained by this study was anonymous. No safety measures were necessary since the participants used the assigned information sources ad libitum.

RESULTS

13a) For each group, the numbers of participants who were randomly assigned, received intended treatment, and were analysed for the primary outcome

NPT: The number of care providers or centers performing the intervention in each group and the number of patients treated by each care provider in each center

Does your paper address CONSORT subitem 13a? *

Copy and paste relevant sections from the manuscript (include quotes in quotation marks "like this" to indicate direct quotes from your manuscript), or elaborate on this item by providing additional information not in the ms, or briefly explain why the item is not applicable/relevant for your study

"The participant flow chart (Figure 1) depicts the subjects who have been enrolled."

13b) For each group, losses and exclusions after randomisation, together with reasons

Does your paper address CONSORT subitem 13b? (NOTE: Preferably, this is shown in a CONSORT flow diagram) *

Copy and paste relevant sections from the manuscript (include quotes in quotation marks "like this" to indicate direct quotes from your manuscript), or elaborate on this item by providing additional information not in the ms, or briefly explain why the item is not applicable/relevant for your study

Reasons for exclusions of participants and lost-to follow up were included in Figure 1.

13b-i) Attrition diagram

Strongly recommended: An attrition diagram (e.g., proportion of participants still logging in or using the intervention/comparator in each group plotted over time, similar to a survival curve) or other figures or tables demonstrating usage/dose/engagement.

subitem not at all important

1 ○

2 ●

3 ○

4 ○

5 ○

essential

Does your paper address subitem 13b-i?

Copy and paste relevant sections from the manuscript or cite the figure number if applicable (include quotes in quotation marks "like this" to indicate direct quotes from your manuscript), or elaborate on this item by providing additional information not in the ms, or briefly explain why the item is not applicable/relevant for your study

Not applicable. The data obtained by this study is anonymous and does not allow the tracking of user activity. Furthermore, we did not regard an attrition diagram as beneficial for our study, because we wanted to gain a deeper understanding about the type and quality of two web-based information sources community pharmacists use for their daily counseling practice in comparison to the EVInews-database.

14a) Dates defining the periods of recruitment and follow-up

Does your paper address CONSORT subitem 14a? *

Copy and paste relevant sections from the manuscript (include quotes in quotation marks "like this" to indicate direct quotes from your manuscript), or elaborate on this item by providing additional information not in the ms, or briefly explain why the item is not applicable/relevant for your study

"[…] online survey with an unblinded search-task and a parallel-study design was carried out between February 1st and April 30th 2021."

14a-i) Indicate if critical "secular events" fell into the study period

Indicate if critical "secular events" fell into the study period, e.g., significant changes in Internet resources available or "changes in computer hardware or Internet delivery resources"

subitem not at all important

1 ○

2 ●

3 ○

4 ○

5 ○

essential

Does your paper address subitem 14a-i?

Copy and paste relevant sections from the manuscript (include quotes in quotation marks "like this" to indicate direct quotes from your manuscript), or elaborate on this item by providing additional information not in the ms, or briefly explain why the item is not applicable/relevant for your study

Not applicable. No special events took place throughout the trial duration.

14b) Why the trial ended or was stopped (early)

Does your paper address CONSORT subitem 14b? *

Copy and paste relevant sections from the manuscript (include quotes in quotation marks "like this" to indicate direct quotes from your manuscript), or elaborate on this item by providing additional information not in the ms, or briefly explain why the item is not applicable/relevant for your study

Not applicable. The study was not terminated earlier.

15) A table showing baseline demographic and clinical characteristics for each group

NPT: When applicable, a description of care providers (case volume, qualification, expertise, etc.) and centers (volume) in each group

Does your paper address CONSORT subitem 15? *

Copy and paste relevant sections from the manuscript (include quotes in quotation marks "like this" to indicate direct quotes from your manuscript), or elaborate on this item by providing additional information not in the ms, or briefly explain why the item is not applicable/relevant for your study

See Table 1. Sociodemographic data of the study sample.

15-i) Report demographics associated with digital divide issues

In ehealth trials it is particularly important to report demographics associated with digital divide issues, such as age, education, gender, social-economic status, computer/Internet/ehealth literacy of the participants, if known.

subitem not at all important

1 ○

2 ○

3 ●

4 ○

5 ○

essential

Does your paper address subitem 15-i? *

Copy and paste relevant sections from the manuscript (include quotes in quotation marks "like this" to indicate direct quotes from your manuscript), or elaborate on this item by providing additional information not in the ms, or briefly explain why the item is not applicable/relevant for your study

See Table 1. Sociodemographic data of the study sample.

16) For each group, number of participants (denominator) included in each analysis and whether the analysis was by original assigned groups

16-i) Report multiple “denominators” and provide definitions

Report multiple “denominators” and provide definitions: Report N’s (and effect sizes) “across a range of study participation [and use] thresholds” [1], e.g., N exposed, N consented, N used more than x times, N used more than y weeks, N participants “used” the intervention/comparator at specific pre-defined time points of interest (in absolute and relative numbers per group). Always clearly define “use” of the intervention.

subitem not at all important

1 ○

2 ○

3 ●

4 ○

5 ○

essential

Does your paper address subitem 16-i? *

Copy and paste relevant sections from the manuscript (include quotes in quotation marks "like this" to indicate direct quotes from your manuscript), or elaborate on this item by providing additional information not in the ms, or briefly explain why the item is not applicable/relevant for your study

We did always provide absolute and when relevant relative numbers. The study groups were defined in the methods section. All proportions of study participants were provided in relation to the total number of participants in each group.
For example:
“A total of 25.0 % (11/44) of participants within the Web-group agreed fully that they were able to find the desired information in contrast to 70.7 % (41/58) of the EVInews-group.”

16-ii) Primary analysis should be intent-to-treat

Primary analysis should be intent-to-treat, secondary analyses could include comparing only “users”, with the appropriate caveats that this is no longer a randomized sample (see 18-i).

subitem not at all important

1 ○

2 ○

3 ○

4 ●

5 ○

essential

Does your paper address subitem 16-ii?

Copy and paste relevant sections from the manuscript (include quotes in quotation marks "like this" to indicate direct quotes from your manuscript), or elaborate on this item by providing additional information not in the ms, or briefly explain why the item is not applicable/relevant for your study

The primary analysis was based on an intention-to-treat analysis. We used “all” participants for our intention-to-treat analysis. In our specific study context, we furthermore needed to examine, whether the distinct difference between the study groups remained, when analyzing the participants who completed the user-task to a certain extent (“completed partially”) and fully (“completed fully”). A subgroup analysis was therefore also conducted.

17a) For each primary and secondary outcome, results for each group, and the estimated effect size and its precision (such as 95% confidence interval)

Does your paper address CONSORT subitem 17a? *

Copy and paste relevant sections from the manuscript (include quotes in quotation marks "like this" to indicate direct quotes from your manuscript), or elaborate on this item by providing additional information not in the ms, or briefly explain why the item is not applicable/relevant for your study

See for example Table 2. All results were presented with the corresponding measures of location and dispersion.
“Results for the primary target parameter (quality score) were expressed as median and first and third quartile (Q_{25}/Q_{75}).”

17a-i) Presentation of process outcomes such as metrics of use and intensity of use

In addition to primary/secondary (clinical) outcomes, the presentation of process outcomes such as metrics of use and intensity of use (dose, exposure) and their operational definitions is critical. This does not only refer to metrics of attrition (13-b) (often a binary variable), but also to more continuous exposure metrics such as “average session length”. These must be accompanied by a technical description how a metric like a “session” is defined (e.g., timeout after idle time) [1] (report under item 6a).

subitem not at all important

1 ○

2 ●

3 ○

4 ○

5 ○

essential

Does your paper address subitem 17a-i?

Copy and paste relevant sections from the manuscript (include quotes in quotation marks "like this" to indicate direct quotes from your manuscript), or elaborate on this item by providing additional information not in the ms, or briefly explain why the item is not applicable/relevant for your study

Not applicable. The participants used their assigned information sources ad libitum. The information retrieval time was automatically assessed through the survey software however. We therefore presented the average retrieval time within the results section.
“The times needed for the search-task completion are shown in Table 4. […] The median for the search-task completion was 25.4 minutes (IQR: 12.7 – 43.5 min.) […].”

17b) For binary outcomes, presentation of both absolute and relative effect sizes is recommended

Does your paper address CONSORT subitem 17b? *

Copy and paste relevant sections from the manuscript (include quotes in quotation marks "like this" to indicate direct quotes from your manuscript), or elaborate on this item by providing additional information not in the ms, or briefly explain why the item is not applicable/relevant for your study

Not applicable.

18) Results of any other analyses performed, including subgroup analyses and adjusted analyses, distinguishing pre-specified from exploratory

Does your paper address CONSORT subitem 18? *

Copy and paste relevant sections from the manuscript (include quotes in quotation marks "like this" to indicate direct quotes from your manuscript), or elaborate on this item by providing additional information not in the ms, or briefly explain why the item is not applicable/relevant for your study

See
“Table 2. Differences in quality scores between study groups based on the rating scheme evaluation” and “Figure 3. Boxplots of quality scores between study groups and subgroups. The whiskers represent the minimum and the maximum of quality scores. The values represent the medians. n= absolute number of participants.”
We also included the results of the subgroup analysis in Table 2 and Figure 3 because of space restrictions. We however clearly defined our primary outcome parameter initially and the results of the subgroup analysis are clearly labelled as such. Correlational analysis was also performed and is presented within the results section.

18-i) Subgroup analysis of comparing only users

A subgroup analysis of comparing only users is not uncommon in ehealth trials, but if done, it must be stressed that this is a self-selected sample and no longer an unbiased sample from a randomized trial (see 16-iii).

subitem not at all important

1 ○

2 ●

3 ○

4 ○

5 ○

essential

Does your paper address subitem 18-i?

Copy and paste relevant sections from the manuscript (include quotes in quotation marks "like this" to indicate direct quotes from your manuscript), or elaborate on this item by providing additional information not in the ms, or briefly explain why the item is not applicable/relevant for your study

See above.
We also included the results of the subgroup analysis in table 2 and Figure 3 because of space restrictions. We however clearly defined our primary outcome parameter initially and the results of the subgroup analysis are clearly labelled as such.

19) All important harms or unintended effects in each group
(for specific guidance see CONSORT for harms)

Does your paper address CONSORT subitem 19? *

Copy and paste relevant sections from the manuscript (include quotes in quotation marks "like this" to indicate direct quotes from your manuscript), or elaborate on this item by providing additional information not in the ms, or briefly explain why the item is not applicable/relevant for your study

We did not observe any harmful or unintended effects in both study groups. The difference in quality scores was surprising and in favor of the EVInews group.

19-i) Include privacy breaches, technical problems

Include privacy breaches, technical problems. This does not only include physical “harm” to participants, but also incidents such as perceived or real privacy breaches [1], technical problems, and other unexpected/unintended incidents. “Unintended effects” also includes unintended positive effects [2].

subitem not at all important

- 1 ○
- 2 ○
- 3 ○
- 4 ●
- 5 ○

essential

Does your paper address subitem 19-i?

Copy and paste relevant sections from the manuscript (include quotes in quotation marks "like this" to indicate direct quotes from your manuscript), or elaborate on this item by providing additional information not in the ms, or briefly explain why the item is not applicable/relevant for your study

We did not observe or experience any privacy breaches as well as technical problems. Participants did also not report any issues of that nature throughout the study duration and beyond.

19-ii) Include qualitative feedback from participants or observations from staff/researchers

Include qualitative feedback from participants or observations from staff/researchers, if available, on strengths and shortcomings of the application, especially if they point to unintended/unexpected effects or uses. This includes (if available) reasons for why people did or did not use the application as intended by the developers.

subitem not at all important

- 1 ○
- 2 ○
- 3 ●
- 4 ○
- 5 ○

essential

Does your paper address subitem 19-ii?

Copy and paste relevant sections from the manuscript (include quotes in quotation marks "like this" to indicate direct quotes from your manuscript), or elaborate on this item by providing additional information not in the ms, or briefly explain why the item is not applicable/relevant for your study

“Rater-performance
The extent of agreement between the 2 raters measured using the Cohen’s kappa statistic was “almost perfect“ (κ 0.81 - 1) for the rating scheme items 1, 2 ,4a ,4d – 13 and 15 – 18. The extent of agreement on the items 14 complied with “substantial agreement” (κ=0.780), the item 3 as well as 4c with “moderate agreement” (κ 0.41 – 0.60) and item 4b with “slight agreement” (κ=0.077). All values were significantly different from zero (P<0.001). “

DISCUSSION

22) Interpretation consistent with results, balancing benefits and harms, and considering other relevant evidence

NPT: In addition, take into account the choice of the comparator, lack of or partial blinding, and unequal expertise of care providers or centers in each group

22-i) Restate study questions and summarize the answers suggested by the data, starting with primary outcomes and process outcomes (use)

Restate study questions and summarize the answers suggested by the data, starting with primary outcomes and process outcomes (use).

subitem not at all important

1 ○
2 ○
3 ○
4 ●
5 ○

essential

Does your paper address subitem 22-i? *

Copy and paste relevant sections from the manuscript (include quotes in quotation marks "like this" to indicate direct quotes from your manuscript), or elaborate on this item by providing additional information not in the ms, or briefly explain why the item is not applicable/relevant for your study

We summarized the study objectives and main results within the first paragraph of the discussion section.
“We found that the median quality score was significantly lower in the Web-group than in the EVInews-group. Additionally, the variability of quality medians in the Web-group was significantly larger and less participants completed the search-task fully. Web-based sources often did not meet standard quality requirements. The time needed for the search-task completion did not reveal any considerable differences between the two groups. The data suggests that community pharmacists struggled to find web-based high-quality, evidence-based information sources for self-medication under everyday-practice conditions.”

22-ii) Highlight unanswered new questions, suggest future research

Highlight unanswered new questions, suggest future research.

subitem not at all important

1 ○

2 ○

3 ●

4 ○

5 ○

essential

Does your paper address subitem 22-ii?

Copy and paste relevant sections from the manuscript (include quotes in quotation marks "like this" to indicate direct quotes from your manuscript), or elaborate on this item by providing additional information not in the ms, or briefly explain why the item is not applicable/relevant for your study

“Taken together, the findings suggest that there is a need for customized information sources for pharmacists.”

20) Trial limitations, addressing sources of potential bias, imprecision, and, if relevant, multiplicity of analyses

20-i) Typical limitations in ehealth trials

Typical limitations in ehealth trials: Participants in ehealth trials are rarely blinded. Ehealth trials often look at a multiplicity of outcomes, increasing risk for a Type I error. Discuss biases due to non-use of the intervention/usability issues, biases through informed consent procedures, unexpected events.

subitem not at all important

1 ○

2 ○

3 ○

4 ○

5 ●

essential

Does your paper address subitem 20-i? *

Copy and paste relevant sections from the manuscript (include quotes in quotation marks "like this" to indicate direct quotes from your manuscript), or elaborate on this item by providing additional information not in the ms, or briefly explain why the item is not applicable/relevant for your study

“**Limitations**
A Hawthorne-Effect may have influenced the participants’ search behaviour in both groups, prompting them to look for the very best evidence in the trial setting. Due to the online format they were, however, not directly observed and therefore this effect probably plays a minor role.

Raters and participants were not blinded. Since the type of information sources the users were allowed to use was predefined, blinding was not possible. In order to minimize subjective evaluation, a quantitative approach for the quality evaluation with objective criteria was chosen to ascertain a robust rating performance.

Only 2 OTC-relevant indications were chosen for the search-task (s. Supplement 1). It remains questionable whether these two indications were adequate for the search-task. Customers’ inquiries regarding the evidence or verification of advertising statements for the treatment of female Androgenetic Alopecia or recurrent Herpes labialis are nonetheless common in everyday counseling practice and were therefore included them in this study.

The exclusion of tertiary print literature was due to technical difficulties when conducting the online survey. However, electronic information sources are frequently used among pharmacists nowadays, especially for the verification of health-related claims.

The Rating Scheme was not validated prior to the survey conduction, hence extensive pretests and pilot tests were performed.”

21) Generalisability (external validity, applicability) of the trial findings

NPT: External validity of the trial findings according to the intervention, comparators, patients, and care providers or centers involved in the trial

21-i) Generalizability to other populations

Generalizability to other populations: In particular, discuss generalizability to a general Internet population, outside of a RCT setting, and general patient population, including applicability of the study results for other organizations

subitem not at all important

- 1 ○
- 2 ○
- 3 ●
- 4 ○
- 5 ○

essential

Does your paper address subitem 21-i?

Copy and paste relevant sections from the manuscript (include quotes in quotation marks "like this" to indicate direct quotes from your manuscript), or elaborate on this item by providing additional information not in the ms, or briefly explain why the item is not applicable/relevant for your study

"The differences in quality scores between the participants could also be explained by personal factors, such as different levels of work experience, knowledge and the skills needed to quickly find suitable external evidence on the internet. Significant differences in sociodemographic data between both groups was not initially existent. According to our correlational analysis, there was a negative […]".
We simulated realistic counseling inquiries in form of a search-task. Furthermore, participants within the web-group used the electronic means they would normally use for everyday counseling practice, since we did not restrict the usage of electronic information means. The trial results were therefore obtained under routine conditions. Participants were also recruited from various regions in Germany (north, west, east, south) and the sociodemographic revealed a broad spectrum of characteristics within both study groups. The external validity of this study is therefore expected to be high. Due to space restrictions and our detailed description of the study conditions, we believe that generalizability did not needed to be further addressed within the discussion section.

21-ii) Discuss if there were elements in the RCT that would be different in a routine
application setting

Discuss if there were elements in the RCT that would be different in a routine application setting (e.g., prompts/reminders, more human involvement, training sessions or other co-interventions) and what impact the omission of these elements could have on use, adoption, or outcomes if the intervention is applied outside of a RCT setting.

subitem not at all important

- 1 (selected)
- 2
- 3
- 4
- 5

essential

Does your paper address subitem 21-ii?

Copy and paste relevant sections from the manuscript (include quotes in quotation marks "like this" to indicate direct quotes from your manuscript), or elaborate on this item by providing additional information not in the ms, or briefly explain why the item is not applicable/relevant for your study

Not applicable. We did conduct our study under routine conditions.

OTHER INFORMATION

23) Registration number and name of trial registry

Does your paper address CONSORT subitem 23? *

Copy and paste relevant sections from the manuscript (include quotes in quotation marks "like this" to indicate direct quotes from your manuscript), or elaborate on this item by providing additional information not in the ms, or briefly explain why the item is not applicable/relevant for your study

The trial was not registered prior to the study conduction.

24) Where the full trial protocol can be accessed, if available

Does your paper address CONSORT subitem 24? *

Cite a Multimedia Appendix, other reference, or copy and paste relevant sections from the manuscript (include quotes in quotation marks "like this" to indicate direct quotes from your manuscript), or elaborate on this item by providing additional information not in the ms, or briefly explain why the item is not applicable/relevant for your study

The trial was not registered prior to the study conduction. Therefore, no a priori protocol is available.

25) Sources of funding and other support (such as supply of drugs), role of funders

Does your paper address CONSORT subitem 25? *

Copy and paste relevant sections from the manuscript (include quotes in quotation marks "like this" to indicate direct quotes from your manuscript), or elaborate on this item by providing additional information not in the ms, or briefly explain why the item is not applicable/relevant for your study

“Third party funding
Jennifer Alexa received third party funding from the ABDA—Federal Union of German Associations of Pharmacists (Berlin, Germany). Matthias Richter received third party funding from the ABDA—Federal Union of German Associations of Pharmacists (Berlin, Germany).”

X27) Conflicts of Interest (not a CONSORT item)

X27-i) State the relation of the study team towards the system being evaluated

In addition to the usual declaration of interests (financial or otherwise), also state the relation of the study team towards the system being evaluated, i.e., state if the authors/evaluators are distinct from or identical with the developers/sponsors of the intervention.

subitem not at all important

- 1 ○
- 2 ○
- 3 ○
- 4 ●
- 5 ○

essential

Does your paper address subitem X27-i?

Copy and paste relevant sections from the manuscript (include quotes in quotation marks "like this" to indicate direct quotes from your manuscript), or elaborate on this item by providing additional information not in the ms, or briefly explain why the item is not applicable/relevant for your study

“Conflicts of interest:
Jennifer Maria Alexa is one of the EVInews newsletter authors and composed newsletters under the guidance of Thilo Bertsche. All authors developed the rating scheme.”

About the CONSORT EHEALTH checklist

As a result of using this checklist, did you make changes in your manuscript? *

- ○ yes, major changes
- ● yes, minor

What were the most important changes you made as a result of using this checklist?

Certain terms and phrases were added.

How much time did you spend on going through the checklist INCLUDING making changes in your manuscript *

8 hours

As a result of using this checklist, do you think your manuscript has improved? *

- [x] yes
- [ ] no
- [ ] Sonstiges:

Would you like to become involved in the CONSORT EHEALTH group?

This would involve for example becoming involved in participating in a workshop and writing an "Explanation and Elaboration" document

- [ ] yes
- [x] no
- [ ] Sonstiges:

Any other comments or questions on CONSORT

Not all items of the CONSORT EHEALTH-checklist were applicable to our study, since our intervention did not consist of a single digital application.

STOP - Save this form as PDF before you click submit

To generate a record that you filled in this form, we recommend to generate a PDF of this page (on a Mac, simply select "print" and then select "print as PDF") before you submit it.

When you submit your (revised) paper to JMIR, please upload the PDF as supplementary file.

Don't worry if some text in the textboxes is cut off, as we still have the complete information in our database. Thank you!

Final step: Click submit !

Click submit so we have your answers in our database!

Senden

Alle Eingaben löschen

Geben Sie niemals Passwörter über Google Formulare weiter.

Dieser Inhalt wurde nicht von Google erstellt und wird von Google auch nicht unterstützt. Missbrauch melden - Nutzungsbedingungen - Datenschutzerklärung

Google Formulare
